# The phylogenomic analysis of the anaphase promoting complex and its targets points to complex and modern-like control of the cell cycle in the last common ancestor of eukaryotes

**DOI:** 10.1186/1471-2148-11-265

**Published:** 2011-09-23

**Authors:** Laura Eme, Aurélie Trilles, David Moreira, Céline Brochier-Armanet

**Affiliations:** 1 Aix-Marseille Université, Laboratoire de Chimie Bactérienne - UPR CNRS 9043, Marseille, France; 2 Unité d'Ecologie, Systématique et Evolution - UMR CNRS 8079, Université Paris-Sud, Orsay, France; 3 Present Address: Université de Lyon; Université Lyon 1; CNRS; UMR5558, Laboratoire de Biométrie et Biologie Evolutive, 43 boulevard du 11 novembre 1918, Villeurbanne, France

**Keywords:** Anaphase Promoting Complex, Cohesin Complex, Phylogeny, Eukaryotes, LECA, Evolution

## Abstract

**Background:**

The Anaphase Promoting Complex or Cyclosome (APC/C) is the largest member of the ubiquitin ligase [E3] family. It plays a crucial role in the control of the cell cycle and cell proliferation by mediating the proteolysis of key components by the proteasome. APC/C is made of a dozen subunits that assemble into a large complex of ~1.5 MDa, which interacts with various cofactors and targets.

**Results:**

Using comparative genomic and phylogenetic approaches, we showed that 24 out of 37 known APC/C subunits, adaptors/co-activators and main targets, were already present in the Last Eukaryotic Common Ancestor (LECA) and were well conserved to a few exceptions in all present-day eukaryotic lineages. The phylogenetic analysis of the 24 components inferred to be present in LECA showed that they contain a reliable phylogenetic signal to reconstruct the phylogeny of the domain Eucarya.

**Conclusions:**

Taken together our analyses indicated that LECA had a complex and highly controlled modern-like cell cycle. Moreover, we showed that, despite what is generally assumed, proteins involved in housekeeping cellular functions may be a good complement to informational genes to study the phylogeny of eukaryotes.

## Background

**Figure 1:**
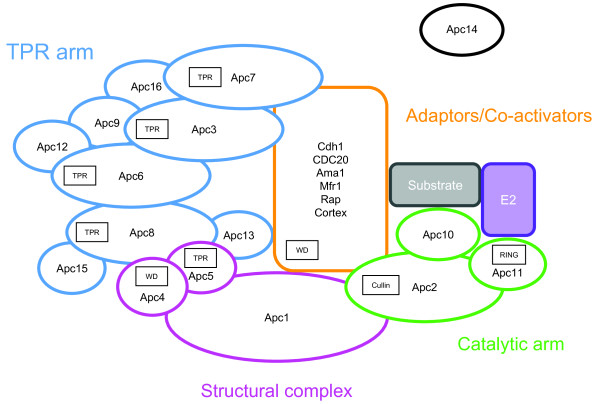
**Schematic structure of the APC/C**. The APC/C is divided in three parts: the structural complex composed of three subunits (in pink), the TPR arm made of nine proteins (in blue) and a second arm (the catalytic arm) made of three proteins (in green) interacting with the E2 protein (in purple) and the substrate (in grey). The activity and specificity of the APC/C is modulated by various adaptor/co-activators (in orange). The location of Apc14 (in black) remains uncertain. The presence of RING finger and Cullin conserved functional domains, and of TPR and WD repeats is indicated.

The anaphase-promoting complex or cyclosome (APC/C) has been recently characterized as a member of the ubiquitin ligase family (also referred as E3) (for a recent review see [1]). E3s mediate the transfer of one or several ubiquitin monomers on a protein substrate in a two-step reaction involving at least three partners. First, an ubiquitin-activating enzyme (E1) activates and transfers ubiquitin to an ubiquitin-conjugating enzyme (E2). Next, E3 mediates the transfer of ubiquitin from E2 to a lysine residue of the target protein. Both steps require ATP. Most E3s are able to polyubiquitinate proteins by adding new ubiquitin monomers to the first attached one [2]. Polyubiquitinated proteins are targeted to the 26S proteasome for degradation, whereas mono-ubiquitinated proteins can be altered in their function or subcellular location by proteins containing ubiquitin-binding domains [2]. E3s are divided in several families according to the presence of signature motifs. Among them, E3s containing a HECT (Homologous to E6-AP Carboxyl Terminus) domain receive ubiquitin from E2 before attaching it on the substrate, whereas E3s harboring a RING (Really Interesting New Gene) finger-domain mediate the transfer of ubiquitin directly from E2 to the substrate [2,3]. RING finger E3s form the largest family and may also contain a subunit with a Cullin domain [1]. Among them, the APC/C is atypically large (1.5 MDa) and complex, being composed of one or several copies of at least a dozen subunits and of various adaptors/co-activators (Figure 1) [4]. The function of the APC/C has been extensively studied in animals and yeast, where it was shown to have a critical role in cell cycle progression through the tight control of degradation of key proteins (e.g. mitotic cyclins, anaphase inhibitors, spindle proteins, regulatory kinases, inhibitors of DNA replication, etc.) [5,6].

Electron microscopy observations, *in vitro *assays, genetic experiments and structural studies have shed light on the composition, structural organization, assembly and molecular activity of the APC/C (Table 1 and Figure 1) [1,4,7]. The APC/C core is divided in three functional parts: i) the structural complex, which is made of Apc1, Apc4 and Apc5 subunits (in pink, Figure 1), serves as scaffold; ii) the catalytic arm (in green, Figure 1) that houses the E2-binding site is made of Apc10 and of the Apc2 and Apc11 proteins that contain the Cullin and RING finger domains, respectively; and iii) the TPR arm (in blue, Figure 1) allows positioning both the E2 and the substrate in order to promote the ubiquitin transfer. This second arm is composed of four subunits (Apc3, Apc6, Apc7, and Apc8) containing tetratricopeptide repeats (TPR) and of several accessory proteins (Apc9, Apc12, Apc13, and Apc15, the last two being essential in *Schizosaccharomyces pombe *[8]). It has been proposed that the TPR repeats interact with the isoleucine- and arginine-rich motifs found in the C-terminal regions of adaptors/co-activators (see [1] and references therein for additional details). The TPR arm may also contain Apc16, a subunit recently reported by the MitoCheck consortium [9]. Finally, the location of Apc14, a yeast essential subunit [8] remains undetermined.

**Table 1:** Taxonomic distribution of homologues of APC/C subunits and adaptors/co-activators in major lineages of eukaryotes.

	Catalytic arm			Structural complex			TPR arm subunits and associated proteins										Mitotic adaptors		Meiotic adaptors		
						
	Apc10	Apc11	Apc2	Apc1	Apc4	Apc5	Apc8	Apc6	Apc3	Apc7	Apc12	Apc13	Apc16	Apc9	Apc15	Apc14	Cdc20	Cdh1	Ama1	Rap	Cortex
Holozoa/ Choanoflagellata	100		100	100	≥50		100	100	100	≥50							100	100			
Holozoa/ Metazoa	100	100	100	100	100	100	100	100	100	100	≥50	≥50	< 50				100	100		< 50	< 50
Holozoa/ Capsaspora	100	100	100	100	100	100	100		100	100							100	100			
Fungi/ Dikarya	100	100	100	100	100	100	100	100	100		≥50	≥50		< 50	< 50	< 50	100	100	≥50		
Fungi/ Microsporidia	100	< 50		100			≥50	100	100								100	100			
Fungi/ Chytridiomycota	≥50	100	100	100	≥50	≥50	100	100	100	100							100	100			
Apusozoa	100	100	100	100	100	100	100	100	100								100	100			
Amoebozoa	100	100	≥50	100	≥50	≥50	≥50	≥50	≥50	≥50	≥50	≥50					≥50	100			
Excavata	≥50	≥50	≥50	≥50			≥50	≥50	≥50	< 50							≥50	< 50			
Alveolata/ Ciliata	100	100	100	100	100		100	100	100								≥50	100			
Alveolata/ Apicomplexa	≥50	≥50		< 50			< 50	< 50	< 50	≥50								≥50			
Heterokonta	100	≥50	≥50	50	< 50	≥50	100	≥50	≥50	≥50	≥50	≥50					100	≥50			
Plantae/ Viridiplantae	100	100	100	100	100	≥50	100	100	100	≥50	< 50	≥50					100	≥50			
Plantae/ Rhodophyta	100	100	100	100			100	100	100								100	100			
Haptophyta	100	100	100	100	100		100	100	100								100	100			

100, > 50, and < 50 indicate that at least one orthologue has been detected respectively in all, at least 50%, and less than 50% of the studied genomes for a given lineage.

The APC/C activity and specificity are modulated by several adaptors/co-activators (Cdc20, Cdh1, Ama1, Mfr1, Rap and Cortex) (Table 1 and Figure 1) [7,10]. These are paralogous proteins containing WD-repeats that mediate the interaction between the APC/C and the D-, KEN-, A- or O-boxes present on target substrates (see [11] and references therein). Among those adaptors, Cdc20 and Cdh1 are the most important, being directly involved in the activation and substrate selectivity of the APC/C at different stages of the cell cycle (reviewed in [1,6,7]). The interaction of the APC/C and either Cdc20 (in early-mid-mitosis) or Cdh1 (during late-mitosis and G1-S transition) is strongly dependent on the high or low activity of Cdks (Cyclin dependent kinases) [6]. Briefly, Cdc20 activates the APC/C during early mitosis once the chromosomes are properly attached and bi-oriented at the metaphase plate during a process known as the spindle assembly checkpoint [12]. The APC/C^-Cdc20 ^targets securins and cyclins B1 towards destruction by the proteasome. The degradation of these two proteins promotes the activation of separases, which then cleave the cohesin complex (CC) leading to the separation of sister chromatids and the initiation of the anaphase [6,12,13]. During anaphase, the APC/C^-Cdh1 ^targets Polo-like kinase 1, Aurora kinases, mitotic cyclins and Cdc20 towards degradation leading to the exit of mitosis. The APC/C^-Cdh1 ^remains active during the G1/S phase ensuring the degradation by the 26S proteasome of several inhibitors of DNA replication, thus allowing the synthesis of DNA [7]. At the end of the S phase, the increase of the activity of Cdks inhibits the interaction between Cdh1 and the APC/C complex, precluding new rounds of DNA synthesis [1]. By contrast, other APC/C activators seem to have more restricted roles: Ama1 is required for sporulation and during the anaphase of meiosis I in budding yeast [7,14]; Mfr1 acts at the end of meiosis II in *S. pombe *[10]; Cortex encodes a putative *Drosophila melanogaster *female meiosis-specific co-activator of the APC/C prior to the metaphase I arrest [15] and, finally, Rap (retina aberrant in pattern) mediates the degradation of cyclins during the development of eye imaginal discs in *D. melanogaster *[16].

**Figure 2:**
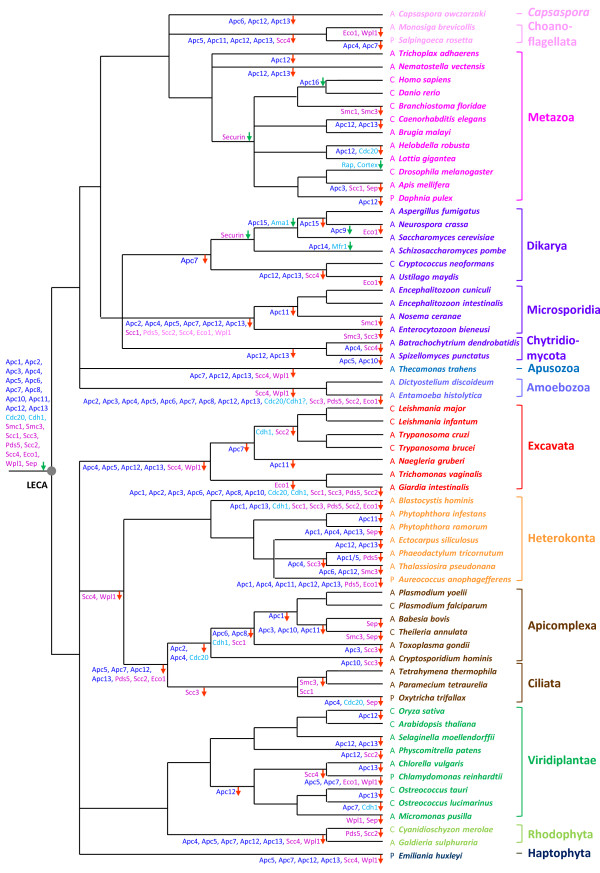
**Evolutionary history of APC/C subunits, adaptors/co-activators and main targets**. We used a reference phylogenetic tree rooted in-between Unikonta and Bikonta [23] showing the relationships between the eleven major eukaryotic lineages for which complete (or nearly complete) genome sequences are available [56,63,64,78]. Holozoa are representated by *Capsaspora*, Choanoflagellata and Metazoa (in pink); Fungi by Dikarya, Chytridiomycota and Microsporidia (in dark purple); Alveolata by Apicomplexa and Ciliata (in brown) and Plantae by Viridiplantae and Rhodophyta (in green). The completion status for each genome sequence is indicated: "C": complete, " A": draft assembly, "P": ongoing (accordingly the loss of some components inferred in the corresponding lineages should be confirmed when complete genome sequences are available). LECA (grey circle) represents the Last Eukaryotic Common Ancestor. Based on phylogenetic analyses, we inferred the time of appearance or loss of orthologues of APC/C subunits (dark blue), activators (light blue) and main targets (pink). For each lineage, protein gains and losses are indicated by green and red arrows, respectively.

If most of the APC/C studies have been carried out in yeast and animals, recent experiments with the land plant *Arabidopsis thaliana *have allowed the identification of 12 transcribed genes that are homologous to vertebrate and yeast APC/C subunits and of eight Cdh1/Cdc20 homologues (Table 1) [17-20]. By contrast, very little information is available for representatives of the other major eukaryotic lineages. The only exception concerns the kinetoplastid species *Trypanosoma brucei*, shown to encode seven APC/C subunit homologues in its genome (Table 1) [21]. The apparent conservation of components of the APC/C in these few distantly related eukaryotes opens the question of the origin and evolution of this atypically large and complex ubiquitin-ligase. To tackle this issue, we carried out an in-depth analysis of 16 APC/C subunits and six adaptors/co-activators (Table 1) in all eukaryotic lineages for which representatives with complete (or nearly complete) genome sequences were available (Figure 2). We also included in our study several major direct or indirect targets of APC/C, namely the separase, the securin, cyclins A and B, Cdks-1 and -2 and the nine components of the cohesin complex (Table 2). The phylogenomic analysis of these proteins supports that most of them were present in the last eukaryotic common ancestor (LECA), indicating that this organism likely possessed a highly controlled cell cycle that may have been very similar to that of present-day eukaryotes. Finally our analyses indicate that APC/C components and targets carry a *bona fide *phylogenetic signal that can be used to trace back the evolutionary history of the eukaryotic domain.

**Table 2:** Taxonomic distribution of homologues of major APC/C targets in major lineages of eukaryotes.

	Core complex				Loading complex		Cohesion establishment	Maintenance		Dissolution	
					
	Smc1	Smc3	Scc1	Scc3	Scc2	Scc4	Eco1	Pds5	Wpl1/Rad61	Separase	Securin
Choanoflagellata	100	100	100	100	100		Half	100	Half	100	
Metazoa	**>**50	**>**50	**>**50	100	100	100	100	100	100	**>**50	< 50
Capsaspora	100	100	100	100	100	100	100	100	100	100	
Fungi/Dikarya	100	100	100	100	100	**>**50	**>**50	100	100	100	< 50
Fungi/Microsporidia	**>**50	**>**50		**>**50						**>**50	
Fungi/Chytridiomycota	100	100	100	100	100	50	100	100	100	100	
Apusozoa	100	100	100	100	100		100			100	
Amoebozoa	100	100	100	**>**50	**>**50		**>**50	**>**50		100	
Excavata	100	100	**>**50	**>**50	< 50		**>**50	**>**50		100	
Alveolata/Ciliata	100	< 50	< 50							**>**50	
Alveolata/Apicomplexa	100	**>**50	< 50	**>**50						**>**50	
Heterokonta	100	**>**50	**>**50	**>**50	**>**50		**>**50	**>**50		**>**50	
Plantae/Viridiplantae	100	100	100	100	**>**50	**>**50	**>**50	100	**>**50	**>**50	
Plantae/Rhodophyta	100	100	100	100	**>**50		100	**>**50		100	
Haptophyta	100	100	100	100	100		100	100		100	

100, > 50, and < 50 indicate that at least one orthologue has been detected respectively in all, at least 50%, and less than 50% of the studied genomes for a given lineage.

## Results and Discussion

### Most APC/C components and main targets were present in LECA

We used a phylogenomic approach [22] in order to study the origin and evolution of APC/C and its main targets in eukaryotes. The first step consisted in the survey of complete (or nearly complete) genome sequences available in public databases to retrieve homologues of each component of this system. Working on complete genomes ensures the rigorous inference of the presence or absence of homologues in each genome. Then, phylogenetic analyses allow inferring the origin and the subsequent evolution of each component.

We searched for orthologues in 65 taxa representing the eukaryotic diversity (all major groups were present except Rhizaria and Cryptophyta). More precisely, our taxonomic sampling covered (i) Holozoa (represented by Metazoa, and their close relatives the Choanoflagellata and *Capsaspora*), (ii) Fungi (with three main lineages: Dikarya, Chytridiomycota and Microsporidia) and (iii) Apusozoa. Whereas the position of Apusozoa remains uncertain, Metazoa, their unicellular allies and Fungi represent the opisthokont lineage that together with Amoebozoa, form one of the two putative major divisions of eukaryotes, the Unikonta [23]. The other major division, the Bikonta, was represented in our study by genomes from Excavata, Heterokonta, Apicomplexa and Ciliata (two main groups within the Alveolata), Haptophyta, and Viridiplantae and Rhodophyta (two main Plantae lineages) (Figure 2).

At this step, it is interesting to notice that, except for adaptors/co-activators and a few other exceptions, we identified at most only one homologue of each APC/C component and main target coding genes in each genome (Additional file 1, Table S1 and Additional file 2, Table S2). In addition, some of them were found only in very restricted sets of species. For example, orthologues of Apc14 and of two subunits of the TPR arm (Apc9 and Apc15) were present only in a few ascomycetes (Table 1 and Additional file 1, Table S1) suggesting that they are recent innovations that emerged after the first diversification of fungi (Figure 2). The TPR arm protein Apc16 may be even more recent because it was present only in the two Gnathostomata representatives within metazoa (Table 1 and Figure 2). By contrast, based on ML and Bayesian phylogenetic analyses (Additional file 3, Data S1), we identified orthologues of the other 12 APC/C subunits and of two adaptors/co-activators in at least two bikont and two unikont major lineages, indicating that they were likely present in LECA (Table 1 and Figure 2). Accordingly, their absence in any present-day genome should be interpreted as secondary loss. This is for instance the case of the six subunits composing the catalytic arm and the structural complex, for which we detected homologues in all opisthokonts but choanoflagellates, which ancestrally missed Apc11 and Apc5, suggesting ancient losses in this lineage. Similarly, whereas the four main subunits of the TPR arm (Apc3, Apc6, Apc7 and Apc8) were inferred to be present in the ancestor of opisthokonts, Apc7 was missing in ascomycete and basidiomycete representatives suggesting a secondary loss in the ancestor of these two fungal groups after their separation from chytrids. Regarding the other proteins associated to the TPR arm, beside the case of Apc9, Apc15 and Apc16 already mentioned, homologues of Apc12 and Apc13 were poorly represented in opisthokonts. More precisely they were missing in choanoflagellates, *Capsaspora*, most fungi and some animals. However, the presence of Apc12 and Apc13 orthologues also in some bikont lineages indicated that these subunits were present in LECA and thus in the ancestor of opisthokonts. Accordingly, their poor taxonomic distribution in this eukaryotic lineage results from convergent secondary losses (Figure 2). Finally, orthologues of the two adaptors Cdc20 and Cdh1 were present in all opisthokonts. Among them, the case of Microsporidia deserved attention. Indeed, whereas only one APC/C subunit (Apc10, located in the catalytic arm) had previously been reported in *Encephalitozoon cuniculi *[24], we additionally found orthologues of one component of the structural complex (Apc1), three of the TPR arm (Apc3, Apc6 and Apc8) and of two adaptors/co-activators (Cdh1 and Cdc20) in genomes of four representatives of this group of highly derived parasitic anaerobic fungi. The conservation of at least one component of each functional part of the APC/C suggested that a minimalist version of the APC/C might exist in Microsporidia. More drastic losses were observed in the anaerobic parasite *Entamoeba histolytica *where the absence of all but four components contrasted with the conservation of all 14 subunits and adaptors/co-activators inferred to be present in LECA in the second amoebozoan studied (the free-living *Dictyostelium discoideum*) (Table 1 and Figure 2). Such massive losses were also observed for the parasitic excavate *Giardia intestinalis*. However, in contrast with Microsporidia, the more reduced set of components and, more precisely, the absence of all proteins composing the TPR arm appeared less compatible with a fully operational APC/C system in these two anaerobic parasites.

In bikonts, orthologues of the 12 components inferred to be present in LECA were also inferred to be present in the ancestors of Plantae and Heterokonta (Figure 2). However, in red algae, the haptophyte *Emiliania huxleyi*, ciliates and most excavates, a slightly more restricted set of proteins was observed (Table 1). Notably, none of them harboured the Apc5 subunit of the structural complex, along with two components of the TPR arm (Apc12 and Apc13), whereas we detected Apc4 only in the ciliate *Tetrahymena thermophila *and the haptophyte *E. huxleyi*, and Apc7 in the excavate *Trichomonas vaginalis *(Table 1). This suggested that the great similarity observed in the distribution of APC/C components in these four major eukaryotic lineages was likely due to convergent losses during their evolution (Figure 2). The situation was completely different in Apicomplexa, the second main lineage of alveolates in our dataset together with ciliates. While the three ciliates have retained a large number of components inferred to be present in LECA (Table 1), most of them have been lost in Apicomplexa (Table 1). More specifically, all APC/C proteins were missing in *Babesia bovis *and *Theileria annulata*, whereas the remaining four apicomplexan species harboured only four or five of them. Surprisingly, components present in those organisms were diverse depending on the species (Table 1). For instance, Apc10, Apc11 and Apc1 were found in *Toxoplasma gondii*, whereas the two *Plasmodium *species contained orthologues of Apc10, Apc11, Apc3 and the anaerobic *Cryptosporidium hominis *harboured Apc1, Apc11, Apc3, Apc6 and Apc8 (Additional file 1, Table S1). The presence of nearly all components in ciliates indicated that massive and differential losses occurred secondarily in Apicomplexa (Figure 2). As mentioned above, such massive losses were also observed in the excavate *G. intestinalis *and in the amoebozoan *E. histolytica *(Additional file 1, Table S1). However, it is important to note that when orthologues existed in these lineages, they showed highly divergent sequences compared to those found in other eukaryotic lineages (not shown). It is thus possible that orthologues of some components might have escaped detection (despite the use of PSI-BLAST searches) because of their extreme degree of sequence divergence. In any case, the possible massive losses or the high divergence of APC/C components both suggested that important changes have occurred relatively recently in these parasitic lineages, maybe as a consequence of their atypical cell division mechanism. This is notably the case of *Theileria *that, acting like a disguised chromosome during host cell division, inserts itself into both daughter cells by co-opting parts of the host cell division machinery, in particular the host cell's microtubules to be pulled towards the opposing ends of the dividing cell [25].

An interesting evolutionary pattern emerged from our analyses concerning the APC/C adaptors/co-activators. Their phylogenies supported that only the two paralogues Cdc20 and Cdh1 were present in LECA and conserved in nearly all eukaryotic lineages (even in Microsporidia), whereas all the remaining co-activators resulted from independent duplications that occurred recently in different eukaryotic lineages (Additional file 4, Figure S1 and Additional file 5, Figure S2). For instance, Rap and Cortex resulted from duplications of Cdh1 and Cdc20, respectively, which occurred in *D. melanogaster*, whereas Ama1 and Mfr1 derived from duplications of Cdc20 in Ascomycota and Cdh1 in *S. pombe*, respectively. Within plants, our analyses confirmed the presence of multiple Cdc20 and Cdh1 copies in *A. thaliana*, but also in other land plants (*Oryza sativa*, *Selaginella moellendorfii *and *Physcomitrella patens*) (Additional file 1, Table S1) [19]. Phylogenetic analyses suggested that the numerous homologues observed in these plants arose from independent duplications (Additional file 4, Figure S1 and Additional file 5, Figure S2). In *A. thaliana*, the various Cdc20 and Cdh1 paralogues have been shown to be differently expressed through the cell cycle and depending on cell types or tissues [19], suggesting that subfunctionalization events occurred after the duplications. More intriguing was the huge expansion of the repertory of adaptor/co-activators observed in the two ciliates *T. thermophila *and *Paramecium tetraurelia*, for which we identified eight and ten copies of Cdh1, respectively. Among excavates, *Leishmania *and *Trypanosoma *genomes encoded only one adaptor/co-activator affiliated to the Cdc20 subfamily (Table 1), whereas the genome of *Naegleria *(as in most eukaryotes) encoded one Cdc20 and one Cdh1 copies. The genome of *Trichomonas *contained three homologues but due to their great divergence we were unable to classify them as Cdc20 or Cdh1 without ambiguity (Additional file 1, Table S1). Finally, in *G. intestinalis *as in some apicomplexa (*Babesia*, *Theileria *and *Toxoplasma*), we failed to detect any adaptor/co-activator, reinforcing the hypothesis that their APC/C proteins have experienced a very divergent and fast evolution.

Regarding the main APC/C targets, our phylogenetic analyses were not conclusive in the case of cyclins A and B and Cdks-1 and -2 to determine whether they were found in LECA or not because these proteins belonged to very large multigenic families with complex evolutionary histories precluding a precise inference of their evolutionary origin (not shown). For the remaining targets, our analyses allowed inferring that the separase and the nine subunits composing the CC (i.e. the core complex, associated proteins, the loading complex and the proteins involved in the cohesion establishment) were present in LECA (Table 2 and Additional file 2, Table S2) and have been conserved in most eukaryotic lineages (Table 2 and Figure 2). The taxonomic distribution of Smc1, Smc3, Scc1, Scc3 and Psd5 homologues was globally in agreement with a previous study focused on the analysis of 29 genes involved in meiosis in eukaryotes [26]. In contrast, Scc4 (a protein composing the loading complex) and Wpl1/Rad61 (a protein that forms together with Psd5 a complex involved in CC maintenance [12,13]) were present only in Viridiplantae and in Opisthokonta suggesting convergent losses in other lineages (Table 2 and Figure 2). However, it can also be speculated that these proteins are not under strong selective pressure, as attested by the fast evolutionary rate of Wpl1/Rad61 found in Saccharomycetaceae that are shorter and highly divergent compared to those of metazoa (647 versus 1190 amino acids, 14% identity and 29% similarity between yeast (NP_010297) and human (NP_055860) sequences). So it would even be possible that they have been replaced by non-homologous proteins in other lineages. The only targets of APC/C that were not inferred to be present in LECA are securins that were found only in Metazoa and Fungi. However, even though they fulfil the same function (preventing the separation of the two sister chromatids) through the binding of separases that are homologous in metazoan and fungal species, fungal securins are not homologous to those from metazoa [27], suggesting again a non-homologous replacement in one of these two groups (Table 1 and Figure 2).

### Functional data point to a nearly modern APC/C controlling the cell cycle in LECA

Our phylogenomic analysis of the APC/C, its main adaptors/co-activators and targets supported the hypothesis that most of the corresponding genes were already present in LECA. It was therefore tempting to conclude that a complex similar to the one involved in present-day regulation of the eukaryotic cell cycle was already present in this ancestral organism. Supporting this hypothesis, nearly all the proteins under study possessed conserved functional domains similar to those present in components that were experimentally proved to be part of the APC/C or targeted by this ubiquitin ligase (Additional file 6, Table S3 and Additional file 7, Table S4). This indicated that all the identified orthologues of APC/C components and targets had similar molecular functions. Moreover, experimental data from plants and excavates have shown that most components identified by our analysis were part of or targeted by the APC/C [18-21,28-35]. This strongly suggested that they inherited their function from the ancestral proteins present in LECA, and therefore that a nearly modern APC/C and control of the cell cycle existed in LECA. This also suggested that, in lineages for which no functional data were available, the orthologues that we have identified were likely involved in the control of the cell cycle and, therefore, may constitute interesting targets for experimental work.

Although the origin of most APC/C components and targets could be traced back to LECA and most of them have been conserved throughout the evolution of eukaryotes, some component gains could also be observed. Most of them resulted from gene duplications of adaptors/co-activators that occurred independently in different lineages. This was in agreement with recent reports of new, and often specific, activities of the APC/C in some eukaryotes (e.g. the neuronal activity [36] or the early eye development [16], or the progression of the endocycle in flowering plant endosperm [18,20]). This suggested that most of the APC/C evolution since LECA has concerned the acquisition of new regulatory functions by increasing the repertoire of adaptors/co-activators, even if we can not rule out the possibility that adaptors/co-activators present in single copies in some lineages were (and still are) able to interact with a larger spectrum of targets than their multiple-copies counterparts. In addition to the classical activators/co-activators, a recent interactomic study in *A. thaliana *suggested the presence of three novel APC/C interactors specific to land plants that were not homologous to the Cdc20/Cdh1 family [34]. However, although they interacted with the APC/C, their biological function (e.g. adaptor/co-activators, inhibitors, targets, etc.) has still not been established. Nevertheless, this supported that lineage-specific innovations are expected to be discovered when biological data on a broader diversity of eukaryotes becomes available.

In contrast, we also observed that convergent events of streamlining occurred secondarily in various lineages, like Apicomplexa, *G. lamblia *and *E. histolytica*. The reasons explaining those massive loss events are unclear, though we could not discard that, at least for a number of cases, they might be linked to an extreme acceleration of their evolutionary rate beyond detection. This hypothesis was supported by the high evolutionary rates exhibited by the few components still harboured by Apicomplexa. However, the detection of APC/C subunits and targets in fast evolving organisms, like *E. cuniculi *and *T. vaginalis*, rather suggested that most of the missing components in *G. lamblia*, *E. histolytica *and Apicomplexa, reflected true losses. In that case we could wonder whether the lost components have been replaced by non homologous proteins that fulfil the same role or whether these parasites are able to recruit the APC/C components from their hosts. In both cases, experimental investigations in these parasitic lineages will be useful to elucidate the nature of their APC/C or even to discover putative divergent systems involved in the control of the cell cycle that may provide interesting medical drug targets. Likewise, a previous phylogenomic study of proteins involved in late cytokinesis revealed a similar pattern of reductive evolution in these lineages [37]. Indeed, *E. histolytica*, *G. lamblia *and Apicomplexa have undergone massive losses of proteins of the cytokinesis machinery, including conserved ancient ones inferred to have been present in LECA [37]. This information combined to our present analysis suggests that major changes have occurred in various steps of the cell cycle in these parasitic eukaryotes.

### Most components of the APC/C and targets are eukaryotic innovations

Despite our extensive survey of public sequence databases, we did not identify any homologue of the APC/C components in prokaryotes with two exceptions. This indicated that this large E3 complex and its main targets are eukaryotic innovations that emerged after the separation of this domain from prokaryotes but prior to its diversification into the present-day eukaryotic lineages. The two exceptions, Smc1 and Smc3, are two paralogous proteins that are part of the core complex of the cohesin complex (Table 1). According to previous reports and to their critical role in higher-order chromosome organization and dynamics [38,39], we identified homologues of Smc1 and Smc3 in nearly all archaeal and bacterial lineages (not shown). The lack of APC/C prokaryotic homologues was surprising because distant homologues harbouring structures similar to eukaryotic ubiquitin, E1 and E2 exist in prokaryotes [40] and because a *bona fide *homologue of the eukaryotic proteasome has been described in Archaea and Actinobacteria [41,42]. Moreover, it was recently reported that homologues of the eukaryotic ubiquitination pathway are encoded in the genome of the archaeon '*Candidatus *Caldiarchaeum subterraneum' [43], a relative of the recently proposed phylum Thaumarchaeota [44]. This system is composed of a cluster of four genes coding for the ubiquitin, E1-like and E2-like enzymes and a small Zn RING finger protein [43]. The first three proteins are much more similar to their eukaryotic counterparts than to the very distant homologues usually found in prokaryotes. Since no *bona fide *homologue of E3 enzymes has been identified in this archaeon, it was proposed that the fourth protein might mediate the ligation of ubiquitin [43]. Despite this recent discovery, our analyses together with data from the literature suggested that the vast majority of APC/C components and main targets were eukaryotic innovations that may have played a role in the emergence and evolution of the complex cell cycle observed in this domain.

### APC/C components and main targets can be used to infer the phylogeny of eukaryotes

Proteins inferred to have been present in LECA are valuable material to reconstruct the characteristics of this ancestral organism (e.g. [37,45-49]). In addition, they can preserve a phylogenetic signal useful to infer the evolutionary history of eukaryotes. Until now, most analyses dedicated to the reconstruction of the eukaryotic phylogeny were based on the analysis of components of informational systems (those involved in the transmission and expression of the genetic information). This was so because most of the genes coding for these proteins present the advantage of being (i) part of monogenic gene families allowing the easy identification of orthologous proteins, (ii) slowly evolving, (iii) ancient and well conserved among life domains, and (iv) rarely exchanged by horizontal gene transfers. Accordingly, they represent first choice material to investigate ancient evolution in all domains of life [50-53]. Phylogenetic studies of the eukaryotic domain did not escape this rule and most of them have been largely based on the phylogenetic analysis of informational proteins (e.g., [51,54-57]). By contrast, most proteins involved in housekeeping functions (operational proteins) are considered to evolve faster than those of informational systems, and thus to be less suitable to study ancient evolution. Moreover, they are often part of large and complex protein families that have experienced numerous gene duplication and loss events during their evolutionary history, meaning that distinguishing between orthologues and paralogues is difficult and requires fastidious preliminary analyses. Consequently, although these proteins are more numerous than informational ones and often of larger size, they have rarely been used to infer the ancient evolution of eukaryotes. Nevertheless, typical datasets based on informational proteins have been shown to be insufficient to robustly infer all the deep nodes of the global eukaryotic phylogenies [58,59]. Increasing the protein sampling is therefore becoming as necessary as increasing the taxonomic sampling in order to fully resolve the phylogeny of eukaryotes, meaning that new useful protein markers have to be found among the conserved operational proteins.

**Figure 3:**
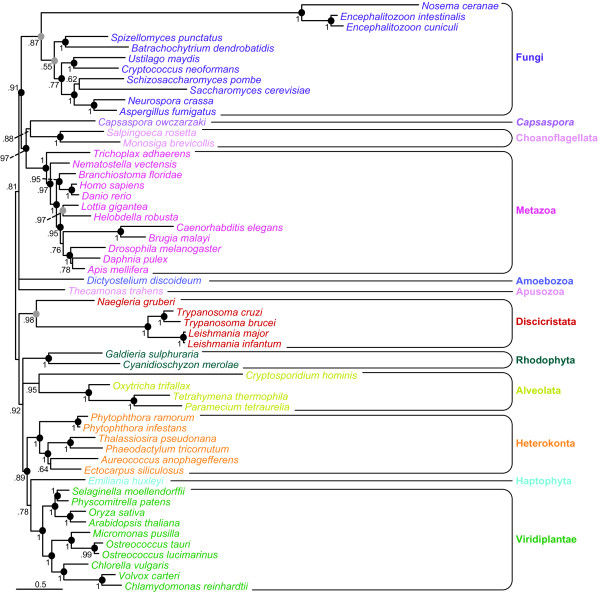
**Bayesian phylogenetic tree of eukaryotes**. The tree was inferred by PhyloBayes based on a supermatrix made of the 24 APC/C components, adaptors/co-activators and main targets inferred to be present in the last common ancestor of eukaryotes (3115 amino-acid positions, 56 species). Numbers at nodes are Bayesian posterior probabilities, filled circles indicate maximum likelihood bootstrap values of 100% (black) or > 95% (grey). The scale bar indicates the average number of substitutions per site.

Our phylogenetic analyses of the APC/C subunits and main targets showed that with a few exceptions they are ancient proteins well conserved throughout the diversity of present day eukaryotic lineages. Accordingly, they are potential suitable markers to reconstruct global eukaryotic phylogenies. The maximum likelihood and Bayesian phylogenetic analyses of individual components showed that they have retained ancient phylogenetic signal despite the fact that some basal nodes of the inferred phylogenies showed a poor resolution (a typical result in single-marker analyses) (Additional file 3, Data S1). To increase resolution, we built a supermatrix (3115 positions) by concatenating the individual alignments of the 24 APC/C components and main targets inferred to be present in LECA. To minimise potential long-branch attraction (LBA) tree reconstruction artefacts [60], we discarded at this step the extremely fast evolving and/or very partial sequences of *Enterocytozoon bieneusi *(Microsporidia), *Plasmodium *spp. (Apicomplexa) and *B. hominis *(Heterokonta), all them having > 60% of missing data. We also discarded *T. vaginalis *(Excavata) because this species contained multiple (and often divergent) copies of several APC/C components and main targets. The resulting Bayesian and maximum likelihood trees recovered the monophyly of each eukaryotic phyla and most often supported by high Bayesian posterior probabilities (PP) and maximum likelihood bootstrap values (BV): Discicristata (PP = 0.98; BV = 97%), Heterokonta (PP = 1.0; BV = 100%), Metazoa (PP = 1.0; BV = 100%), Fungi (PP = 0.87; BV = 98%), Choanoflagellata (PP = 1.0; BV = 100%), Viridiplantae (PP = 1.0; BV = 99%) and Alveolata (PP = 0.95 and BV = 78%) (Figure 3). The most remarkable result was the good support retrieved for the monophyly of Fungi, including the extremely fast evolving Microsporidia. The APC/C proteins are therefore among the rare ones [61] that support the correct phylogenetic placement of these parasites as members of the Fungi.

Regarding the relationships among the main eukaryotic groups, the sister-grouping of the two choanoflagellates and *C. owczarzaki *was supported both by the Bayesian (PP = 0.88) and maximum likelihood (BV = 87%) trees; these two groups being closely related to Metazoa (namely, a support for the Holozoa with PP = 0.97 and BV = 100%). This was in agreement with previous results [62,63], but not with those supporting the emergence of *Capsaspora *at the base of the group composed of Choanoflagellata and Metazoa [64,65]. The phylogenetic signal contained in APC/C components and main targets also supported the monophyly of Opisthokonta (PP = 0.91; BV = 100%). Moreover, if we accept the putative unikont-bikont rooting [23], our analyses also retrieved the Unikonta, including the apusozoan *Thecamonas trahens*, albeit with a weaker support (PP = 0.81; BV = 78%). Similarly, most of the relationships among bikont lineages were poorly resolved (Figure 3). In particular, the monophyly of Plantae was not recovered: green plants and algae appeared closely related to the haptophyte *E. huxleyi *(PP = 0.78; BV = 81%), whereas the phylogenetic position of red algae was not resolved. This agreed with other studies showing the difficulty to retrieve the monophyly of Plantae, even with much larger datasets (e.g., [66]). Finally, heterokonts formed the sister group of green plants and haptophytes (PP = 0.89; BV = 100%).

Taken together, our phylogenetic analyses showed that despite the relative small size of the supermatrix constructed with the APC/C components and main targets (3115 positions), it contained an interesting phylogenetic signal that can be useful to infer part of the ancient evolutionary history of eukaryotes. However, the large size of most of the APC/C subunits and main targets made difficult the use of the abundant body of sequence data coming from the analysis of expressed sequence tags (ESTs) because of their incomplete sequence coverage, which limited our taxonomic sampling. Nevertheless, our dataset can now be employed as a reference to enrich it with assembled EST sequences as well as with those from upcoming complete genome sequences from various protist species. This limitation will likely apply to other operational proteins candidates to be used as markers for the reconstruction of global eukaryotic phylogenies but, as already discussed, their inclusion in multi-marker analyses becomes crucial to infer several deep nodes that the traditional supermatrices of informational proteins have failed to resolve.

## Conclusions

Our analyses of the APC/C and its main targets showed that this complex system was very likely present in LECA and has been conserved, to a few exceptions, all along the diversification of the eukaryotic domain. This study provided first insights into the mechanisms responsible of the control of the cell cycle in LECA, suggesting that it was tightly regulated like in present-day eukaryotes. Finally we showed that the components of the APC/C and its main targets can be good phylogenetic markers to complement those used so far (mostly proteins involved in transcription and translation). Indeed, the latter have proven not to be sufficient to fully resolve the phylogeny of eukaryotes, making necessary to identify new complementary markers. This will certainly be a difficult task that will require many analyses but we think that the phylogenomic study of conserved cellular systems is a promising approach to tackle this issue.

## Methods

### Dataset assembly

We used the 37 APC/C components and main targets identified in four opisthokont species (*Homo sapiens*, *D. melanogaster*, *S. cerevisiae *and *S. pombe *[7,11]), the plant *A. thaliana *[17,19] and the kinetoplastid *T. brucei *[21] (Table 1) to survey public sequence databases. We identified homologues of these proteins using BLASTp and PSI-BLAST (with default parameters) [67] in a subset of complete or ongoing genomes representative of eukaryotic diversity available at the NCBI (Figure 2). To increase the taxonomic sampling, homologues of *Monosiga brevicollis*, *Salpingoeca rosetta*, *Lottia gigantea*, *Nematostella vectensis*, *Helobdella robusta*, *Daphnia pulex*, *Capsaspora owczarzaki*, *Batrachochytrium dendrobatidis*, *Spizellomyces punctatus*, *Thecamonas trahens*, *Naegleria gruberi*, *Phaeodactylum tricornutum*, *Aureococcus anophagefferens*, *Ostreococcus lucimarinus*, *Physcomitrella patens*, *Chlorella vulgaris*, *Micromonas pusilla*, *Selaginella moellendorffii *and *Emiliania huxleyi *were retrieved using the BLASTp and tBLASTn programs from the JGI http://genome.jgi-psf.org/, the Broad Institute http://www.broadinstitute.org and the TBestDB database http://tbestdb.bcm.umontreal.ca/[68]. In addition, homologues of two representatives of Rhodophyta were retrieved from the *Galdieria sulphuraria *genome project http://genomics.msu.edu/cgi-bin/galdieria/blast.cgi and the *Cyanidioschyzon merolae *genome project http://merolae.biol.s.u-tokyo.ac.jp/blast/blast.html. BLAST outputs were examined by eye to identify homologues of each protein to avoid applying an arbitrary cutoff on e-value or score. To ensure an exhaustive sampling of homologues, we performed additional searches using as seeds homologues that were identified at previous steps. The absence of any homologue in a given lineage was systematically verified by hand using tBLASTn searches on the nucleotide sequences of the corresponding complete genomes.

For each protein, the homologous sequences were gathered in a dataset and aligned with MAFFT 6.833 [69]. All the resulting alignments were edited and manually adjusted using the ED program from the MUST package [70]. Regions where homology between sites was doubtful were manually removed from the alignments before phylogenetic analyses. A supermatrix was built by concatenating the alignments corresponding to the 24 APC/C components, adaptor/co-activators and targets inferred to be present in LECA.

### Conserved functional domain search

The identification of functional domains was carried out using the HMMER package [71] and the HMM profiles of the Pfam database [72]. HMM profiles having e-values lower than 0.1 were considered as significant.

### Phylogenetic analysis

Phylogenetic trees were reconstructed for each single protein dataset, except for Apc9, Apc14, Apc15, Apc16 and securin because of their very restricted number of homologues. Maximum Likelihood (ML) phylogenetic trees were computed using Treefinder [73] with the LG model [74] and a gamma correction to take into account the heterogeneity of evolutionary rates across sites (4 discrete classes of sites and estimated alpha parameter), as proposed by the model selection tool implemented in Treefinder. The branch robustness of ML trees was estimated using the non parametric bootstrap procedure (100 replicates of the original alignment) implemented in Treefinder with the same parameters than for ML tree inference. Bayesian trees were computed using MrBAYES 3.1.2 [75] with a mixed amino acid substitution model and a gamma correction (four rate categories). The Markov chain Monte Carlo search was run with 4 chains for 1, 000, 000 generations, with trees being sampled every 100 generations (the first 2, 500 trees were discarded as ''burn-in''). Individual Bayesian trees are provided as Additional file 3, Data S1.

TreeFinder and PhyloBayes 3.2 [76] were used to perform ML and Bayesian analyses on the concatenated dataset. Treefinder was run with the same parameters than for the analysis of single datasets. Phylobayes was run using the CAT model and a gamma correction with 4 rate categories [77]. For each dataset, two independent chains were run for at least 10000 cycles, saving one tree in ten. The first 300 trees were discarded as "burn-in", and the remaining trees from each chain in each dataset were used to test for convergence and compute the 50% majority rule consensus tree.

### Inference of the origin of APC/C components and main targets

The phylogenetic analysis of each individual component allowed distinguishing orthologues that originated from speciation events from paralogues that resulted from gene duplications. This point is important because inferring the evolutionary origin of a protein requires the analysis of the evolutionary history of orthologues. To determine the origin of each component and subunit, we used a parsimony criterion, making the assumption that horizontal gene transfers between eukaryotes are rare. Accordingly, the ancestral absence of a component in the ancestor of a taxonomic group was inferred if no orthologues are found in any present-day representative of the group (even in those that were not included in our taxonomic sampling). By contrast, the presence of orthologues in representatives of two lineages was interpreted as the existence of the corresponding component in their last common ancestor. Similarly, the presence of a component in an ancestral taxon was also inferred if orthologues are present in at least one representative of its offspring and in one of its ancestors.

## Authors' contributions

LE, DM and CBA designed the analyses and wrote the paper. LE and AT performed the analyses. All authors read and approved the final manuscript.

## Supplementary Material

Additional file 1 **Table S1**. Table showing the taxonomic distribution of homologues of APC/C subunits and activators in eukaryotes. Click here for file

Additional file 2 **Table S2**. Table showing the taxonomic distribution of homologues of APC/C main targets in eukaryotes. Click here for file

Additional file 3 **Data S1**. Bayesian phylogenetic trees inferred MrBayes (*.con files) and ML phylogenetic trees inferred with TreeFinder (*.tre files = ML topologies; *.boot.trees = topologies generated by the bootstrap analysis) for each APC/C subunit, activator and main target (except Apc9, Apc14, Apc15 and Apc16 due to a too restricted number of homologues). Click here for file

Additional file 4 **Figure S1**. Bayesian phylogenetic tree of the adaptor/co-activator Cdh1 inferred with Mrbayes. Numbers at nodes correspond to posterior probabilities (PP) estimated by MrBayes and bootstrap values (BV) estimated by TreeFinder (only PP and BV greater than 0.5 and 50%, respectively, are shown). The scale bar indicates the average number of substitutions per site. Click here for file

Additional file 5 **Figure S2**. Bayesian phylogenetic tree of the adaptor/co-activator Cdc20 inferred with Mrbayes. Numbers at nodes correspond to posterior probabilities (PP) estimated by MrBayes and bootstrap values (BV) estimated by TreeFinder (only PP and BV greater than 0.5 and 50%, respectively, are shown). The scale bar indicates the average number of substitutions per site. Click here for file

Additional file 6 **Table S3**. Table showing the conserved functional domains present in homologues of APC/C subunits and activators. Click here for file

Additional file 7 **Table S4**. Table showing the conserved functional domains present in homologues of APC/C main targets. Click here for file
