# Supplementary material for: The phylogenomic analysis of the anaphase promoting complex and its targets points to complex and modern-like control of the cell cycle in the last common ancestor of eukaryotes
Source: BMC Evol Biol. 2011 Sep 23;11:265. doi: 10.1186/1471-2148-11-265 (PMC3195147; doi:10.1186/1471-2148-11-265)
Supplement: Additional file 1 — Table S1. Table showing the taxonomic distribution of homologues of APC/C subunits and activators in eukaryotes. [file 1471-2148-11-265-S1.PDF]

Supplementary Table S1. Taxonomic distribution of orthologues of APC subunits and co-activators. Accession numbers are indicated. Acc Num NA indicates that the accession number of the corresponding sequence is not available. Question marks indicate the presence of highly divergent sequences, however based on sequence comparison only it was not possible to determine if they represent homologues.

			Core subunits																Co-activators						
			Catalytic arm			Structural complex			TPR arm and associated proteins									Unknown	Mitosis co-activators			Meiotic co-activator			
			Apc10	Apc11	Apc2	Apc1	Apc4	Apc5	Apc8	Apc6	Apc3	Apc7	Apc12	Apc13	Apc16	Apc9	Apc15	Apc14	CDC20	CDH1	uncertain	Mfr1	Ama1	Rap	Cortex
Opisthokonta	Choanoflagellata	***Monosiga brevicollis***	XP_001746604		XP_001749209	XP_001742225	XP_001744221		XP_001747393	XP_001745786	XP_001747295	XP_001744593							XP_001747841	Acc Num NA					
		Salpingoeca rosetta	Acc Num NA		Acc Num NA	Acc Num NA			Acc Num NA	Acc Num NA	Acc Num NA								Acc Num NA	Acc Num NA					
	Metazoa	***Trichoplax adhaerens***	XP_002109549	XP_002115875	XP_002114958	XP_002109033	XP_002111892	XP_002109398	XP_002109327	XP_002115543	XP_002110345	XP_002107763		XP_002115489					XP_002114324	XP_002114232					
		Homo sapiens	NP_055700	NP_057560	NP_037498	NP_073153	NP_037499	NP_057321	AAC70920	NP_001072113	EAW57688	Q9UJX3	NP_644815	NP_056206	NP_775744				NP_001246	NP_057347					
		Danio rerio	XP_001923028	NP_001091950	CAX13097	CAX12586	NP_001008589	NP_955916	NP_957227	ABC87083	NP_958857	NP_001107057	NP_001004005	XP_697958	NP_001017878				CAN88634	XP_001922908<br>NP_956547					
		Branchiostoma floridae	XP_002598258	XP_002606314	XP_002600222	XP_002610275	XP_002609002	XP_002610040	XP_002590511	XP_002603477	XP_002598631	XP_002605850	XP_002590834	XP_002590107					AAO85336	XP_002603872					
		Drosophila melanogaster	NP_611223	NP_001097122	NP_611862	NP_573025	NP_572522	AAM51081	NP_610036	NP_477397	NP_648093<br>AAA57340	AAL39766	NP_609940	NP_001033954					AAQ23567	NP_611854				NP_524852	NP_523494
		Lottia gigantea	Acc Num NA	Acc Num NA	Acc Num NA	Acc Num NA	Acc Num NA	Acc Num NA	Acc Num NA	Acc Num NA	Acc Num NA	Acc Num NA	Acc Num NA	Acc Num NA					Acc Num NA	Acc Num NA					
		Apis mellifera	XP_396738	XP_001122138	XP_395411	XP_001122449	XP_393301	XP_393771	XP_396943	XP_623316	Acc Num NA	XP_396165	XP_001122028	XP_001120410					XP_624567	XP_623291					
		Caenorhabditis elegans	NP_001021777<br>NP_505679	XP_777934<br>NP_497937	NP_498762	NP_496383	NP_499074	NP_499485	NP_497203	NP_495712	NP_001021714	NP_500116							NP_495051	NP_496075					
		Brugia malayi	XP_001901978	XP_001901276<br>XP_001898974	XP_001894849	XP_001893638	XP_001900733	XP_001893623	XP_001900333	XP_001900464	XP_001898600	XP_001896953	XP_001898246	XP_001897845					XP_001893850	XP_001899995					
		Nematostella vectensis	XP_001630621	XP_001628272	XP_001625156	XP_001632264	XP_001624909	XP_001626465	XP_001635295	XP_001637375	XP_001635851	XP_001633228							XP_001625800<br>XP_001623504	XP_001637197					
		Helobdella robusta	Acc Num NA	Acc Num NA	Acc Num NA	Acc Num NA	Acc Num NA	Acc Num NA	Acc Num NA	Acc Num NA	Acc Num NA	Acc Num NA		Acc Num NA						Acc Num NA					
		Daphnia pulex	Acc Num NA	Acc Num NA	Acc Num NA	Acc Num NA	Acc Num NA	Acc Num NA	Acc Num NA	Acc Num NA	Acc Num NA	Acc Num NA		Acc Num NA					Acc Num NA	Acc Num NA					
	Capsaspora	***Capsaspora owczarzaki***	Acc Num NA	Acc Num NA	Acc Num NA	Acc Num NA	Acc Num NA	Acc Num NA	Acc Num NA		Acc Num NA	Acc Num NA							Acc Num NA	Acc Num NA					
	Fungi	***Cryptococcus neoformans***	XP_571694	XP_777934	XP_568721	XP_570920	XP_572593	XP_776363	XP_569792	XP_570972	XP_566728								XP_572594	XP_572082					
		Ustilago maydis	XP_756827	XM_753342	XP_759373	XP_758574	XP_761900	XP_756294	XP_756798	XP_760206	XP_758236								XP_757794	XP_760064					
		Aspergillus fumigatus	XP_752943	XP_746794	XP_749651	EDP53140	XP_752937	EDP53116	XP_748098	EDP51110	XP_755196		XP_747203	XP_753351					EDP56709	XP_754786			XP_751769		
		Schizosaccharomyces pombe	NP_595803	NP_593423	NP_001018806	NP_595158	XP_001713100	XP_001713082	NP_593300	NP_593301	CAA30532		NP_593643	NP_595754			NP_595636	NP_594611	NP_593161	NP_594674		NP_595081	NP_588462<br>NP_592834		
		Saccharomyces cerevisiae	1GQP_A	NP_010276	EEU06984	EDN62645	NP_010403	EDV10820	NP_012036	CAY81061	CAY77700		NP_116694	NP_010546		NP_013203	NP_012291		NP_011399	EDN61598			NP_011741		
		Neurospora crassa	CAC28560	XP_001728250	XP_964316	XP_959459	XP_956179	XP_964327	XP_961540	CAC18281	XP_956598		XP_964014	XP_960666					XP_965019	CAE76124			XP_956209		
		Encephalitozoon cuniculi	NP_597441			NP_586231			NP_585899	NP_585771	NP_586422								NP_584809	NP_597660					
		Enterocytozoon bieneusi H348	XP_002649405	XP_001827913 (?)		XP_002649879				XP_002650349	XP_002650295								XP_002649951	XP_002650580					
		Nosema ceranae BRL01	XP_002995625			XP_002996689			XP_002995905	XP_002995208	XP_002995969								XP_002995258	XP_002996735					
		Encephalitozoon intestinalis ATCC 50506	XP_003072909			XP_003073796			XP_003073104	XP_003072974	XP_003073964								XP_003072772	XP_003072646					
		Batrachochytrium dendrobatidis	Acc Num NA	Acc Num NA	Acc Num NA	Acc Num NA		Acc Num NA	Acc Num NA	Acc Num NA	Acc Num NA	Acc Num NA							Acc Num NA	Acc Num NA					
		Spizellomyces punctatus		Acc Num NA	Acc Num NA	Acc Num NA	Acc Num NA		Acc Num NA	Acc Num NA	Acc Num NA	Acc Num NA							Acc Num NA<br>Acc Num NA	Acc Num NA<br>Acc Num NA					
Apusozoa		***Thecamonas trahens***	Acc Num NA	Acc Num NA	Acc Num NA	Acc Num NA	Acc Num NA	Acc Num NA	Acc Num NA	Acc Num NA	Acc Num NA								Acc Num NA	XP_002114232					
Amoebozoa		***Dictyostelium discoideum***	XP_646955	XP_637455	XP_643183	XP_638254	XP_638336	XP_641250	XP_644962	XP_001134535	XP_636853	XP_629638	XP_629236	XP_642090					XP_638150	XP_637334					
		Entamoeba histolytica	XP_654652	XP_651657		XP_648466															XP_657064				
Excavata	Metamonada	***Giardia intestinalis***		EET00603																					
		Trichomonas vaginalis	XP_001313595<br>XP_001579634	XP_001325785	XP_001306780	XP_001326479			XP_001327110	XP_001583340<br>XP_001579241	XP_001312985	XP_001316770<br>XP_001581999									XP_001320233<br>XP_001310738<br>XP_001306976				
	Euglenozoa	***Leishmania major***	XP_888563	XP_843556	XP_843480	XP_001684803			XP_001681666	XP_001684662	XP_001687518								XP_001683689						
		Leishmania infantum	XP_001462876	XP_001469259	XP_001469183	XP_001467045			XP_001463991	XP_001466919	XP_001463004								XP_001463634						
		Trypanosoma cruzi	XP_811195	XP_813702	XP_821644	XP_811561			XP_821942	XP_811257	XP_809564								XP_819329						
		Trypanosoma brucei	XP_828039	XP_827311	XP_827412	XP_845449			XP_001219085	XP_845336	XP_823254								XP_847480						
	Heterolobosoa	***Naegleria gruberi***	EFC45514		EFC48750	EFC50559			EFC45000	EFC50413	EFC50492								EFC37108	EFC42486					
Alveolata	Ciliata	***Tetrahymena thermophila***	XP_001028080	XP_001014455	XP_001031380	XP_001031504	XP_001021620		XP_001011597	XP_001026497	XP_001032153								XP_001023872	XP_001031406<br>XP_001028072<br>XP_001012331<br>XP_001017096<br>XP_001033107<br>XP_001030793<br>XP_001010332<br>XP_001024950					
		Paramecium tetraurelia	XP_001453141<br>XP_001450294	XP_001433857	XP_001426605	Acc Num NA	XP_001458727		XP_001425074	XP_001456952	XP_001434341<br>XP_001430646<br>XP_001460999<br>XP_001461175								XP_001428021	XP_001456512<br>XP_001434711<br>XP_001423825<br>XP_001462391<br>XP_001448596<br>XP_001460408<br>XP_001452380<br>XP_001449473<br>XP_001431487<br>XP_001446793					
		Oxytricha trifallax	Acc Num NA	Acc Num NA	Acc Num NA	Acc Num NA			Acc Num NA	Acc Num NA	Acc Num NA									Acc Num NA					
	Apicomplexa	***Plasmodium yoelii***	XP_725062	XP_724743							XP_724142 ?										XP_728399				
		Plasmodium falciparum	XP_001350578	XP_966227							XP_001351576 ?										XP_001347545				
		Cryptosporidium hominis		XP_668394		XP_668328			XP_668598	XP_667497 ?	XP_668239 ?									XP_665894					
		Babesia bovis																							
		Theileria annulata																							
		Toxoplasma gondii	XP_002367626	XP_002368820		XP_002371936																			
Heterokonta	Blastocystae	***Blastocystis hominis***	CBK23071						CBK23690	Acc Num NA									Acc Num NA						
	Phaeophyceae	***Ectocarpus siliculosus***	Acc Num NA	Acc Num NA	Acc Num NA	Acc Num NA	Acc Num NA	Acc Num NA	Acc Num NA	Acc Num NA	Acc Num NA	Acc Num NA							Acc Num NA<br>Acc Num NA	Acc Num NA					
	Oomycota	***Phytophthora ramorum***	Acc Num NA	Acc Num NA	Acc Num NA			Acc Num NA	Acc Num NA	Acc Num NA	Acc Num NA	Acc Num NA	Acc Num NA						Acc Num NA	Acc Num NA					
		Phytophthora infestans	EEY65500		EEY69744	EEY66226	EEY60933	EEY59982	EEY68460	EEY64266	EEY65802	EEY63812	EEY58244	EEY70446					EEY53557	EEY64288					
	Bacillariophyta	***Thalassiosira pseudonana***	XP_002290626	XP_002293598	XP_002291443	XP_002292917		XP_002294732	XP_002291008		XP_002287562	XP_002292854		XP_002290825					XP_002293959<br>XP_002287004	XP_002290622<br>XP_002293663					
		Phaeodactylum tricornutum	XP_002182838	XP_002186283	XP_002181491				XP_002182840	XP_002176654	XP_002176724	XP_002177784	XP_002177671	XP_002177799					XP_002180582<br>XP_002176387	XP_002179858					
		Aureococcus anophagefferens	Acc Num NA		Acc Num NA			Acc Num NA	Acc Num NA	Acc Num NA	Acc Num NA	Acc Num NA							Acc Num NA	Acc Num NA					
Plantae	Viridiplantae	***Oryza sativa***	AAU10698	NP_001049871	BAG91185	EEE63377	EEC74136	EEE53648	NP_001047624	EEE58661	EAZ01716	AAU44035		NP_001060376					EEE69236<br>CAE03470<br>EEE57631	NP_001045543<br>NP_001048804					
		Arabidopsis thaliana	NP_565433	AAL13436	NP_178543	NP_196175	NP_193884	NP_172146	NP_190398	NP_565188	NP_849994<br>NP_188253	NP_850309	AAN10198	NP_565057					NP_568505<br>AAB61049<br>NP_198109<br>NP_195053<br>NP_198060	NP_196888<br>NP_194022<br>NP_192929					
		Chlamydomonas reinhardtii	XP_001693736	XP_001692946	XP_001698597	XP_001693798	XP_001694053		XP_001698877	XP_001693404	XP_001701084			XP_001691845					XP_001695197	XP_001697615					
		Ostreococcus tauri	AAV68616	AAV68620	AAV68613	AAV68612	CAL52461	AAV68614	CAL54508	AAV68615	CAL53302	AAV68618							AAV68609	CAL56578					
		Ostreococcus lucimarinus	XP_001420017	XP_001420379	XP_001420260	XP_001418248	XP_001416348	XP_001418321	XP_001418934	XP_001415756	XP_001418358			XP_001416938					XP_001417356						
		Physcomitrella patens	XP_001760400	XP_001761005	XP_001758242	XP_001770276	XP_001774147	XP_001775896	XP_001765887	XP_001784365	XP_001771285<br>XP_001758114	XP_001753812		XP_001767480					XP_001757257<br>XP_001775446<br>XP_001766400<br>XP_001753296<br>XP_001765142	XP_001769243<br>XP_001774759<br>XP_001769827<br>XP_001773274					
		Chlorella vulgaris	Acc Num NA	Acc Num NA	Acc Num NA	Acc Num NA	Acc Num NA	Acc Num NA	Acc Num NA	Acc Num NA	Acc Num NA								Acc Num NA	Acc Num NA					
		Micromonas pusilla	EEH60930	EEH54465	EEH54093	EEH56332	EEH58620	EEH53809	EEH57771	EEH59359	EEH56226	EEH57219		EEH55073					EEH56365	EEH57236					
		Selaginella moellendorffii	Acc Num NA	Acc Num NA	Acc Num NA	Acc Num NA	Acc Num NA	Acc Num NA	Acc Num NA	Acc Num NA	Acc Num NA	Acc Num NA							Acc Num NA<br>Acc Num NA<br>Acc Num NA	Acc Num NA					
	Rhodophyta	***Cyanidioschyzon merolae***	Acc Num NA	Acc Num NA	Acc Num NA	Acc Num NA			Acc Num NA	Acc Num NA	Acc Num NA								Acc Num NA	Acc Num NA					
		Galdieria sulphuraria	Acc Num NA	Acc Num NA	Acc Num NA	Acc Num NA			Acc Num NA	Acc Num NA	Acc Num NA								Acc Num NA	Acc Num NA					
Haptophyta		***Emiliania huxleyi***	Acc Num NA	Acc Num NA	Acc Num NA	Acc Num NA	Acc Num NA		Acc Num NA	Acc Num NA	Acc Num NA								Acc Num NA	Acc Num NA					
