# Supplementary material for: The phylogenomic analysis of the anaphase promoting complex and its targets points to complex and modern-like control of the cell cycle in the last common ancestor of eukaryotes
Source: BMC Evol Biol. 2011 Sep 23;11:265. doi: 10.1186/1471-2148-11-265 (PMC3195147; doi:10.1186/1471-2148-11-265)
Supplement: Additional file 2 — Table S2. Table showing the taxonomic distribution of homologues of APC/C main targets in eukaryotes. [file 1471-2148-11-265-S2.PDF]

**Supplementary Table S2.** Taxonomic distribution of orthologues of APC/C main targets. Accession numbers are indicated. Acc Num NA indicates that the accession number of the corresponding sequence is not available. Question marks indicate the presence of highly divergent sequences, however based on sequence comparison only it was not possible to determine if they represent homologues.

| | | | Core complex | | | | Loading complex | | Cohesion establishment | Maintenance | | Dissolution | |
|---|---|---|---|---|---|---|---|---|---|---|---|---|---|
| | | | **Smc1** | **Smc3** | **Scc1** | **Scc3** | **Scc2** | **Scc4** | **Eco1** | **Pds5** | **Wpl1/Rad61** | **Separase** | **Securin** |
| **Opisthokonta** | **Choanoflagellata** | ***Monosiga brevicollis*** | Acc Num NA | XP_001746007 | XP_001743156 | XP_001744374 | XP_001749937 | | | XP_001745460 | | XP_001747241 | |
| | | ***Salpingoeca rosetta*** | Acc Num NA | Acc Num NA | Acc Num NA | Acc Num NA | Acc Num NA | | Acc Num NA | Acc Num NA | Acc Num NA | Acc Num NA | |
| | **Metazoa** | ***Trichoplax adhaerens*** | Acc Num NA | XP_002116802 | Acc Num NA | XP_002111813 | XP_002114243 | Acc Num NA | XP_002110319 | XP_002112303 | XP_002112136 | ? | |
| | | ***Homo sapiens*** | NP_006297 | NP_005436 | NP_001130038 | NP_006594<br>NP_005853<br>NP_036579 | DAA05331 | NP_056144 | NP_443143<br>NP_001017420 | NP_001093870 | NP_055860 | NP_036423 | NP_004210 |
| | | ***Danio rerio*** | AAT68048 | NP_999854 | NP_955889 | XP_686812<br>XP_692954<br>CAQ15056 | XP_001920203 | XP_001334031 | XP_002663315<br>NP_001003872 | AAH55602 | XP_688605 | XP_001337869 | XP_695066 |
| | | ***Branchiostoma floridae*** | | | XP_002602455 | XP_002603307 | XP_002587265 | XP_002609698 | XP_002586734 | XP_002611624 | XP_002610333 | XP_002607627 | |
| | | ***Drosophila melanogaster*** | NP_651211 | NP_523374 | AF186473_1 | CAA74654 | ABX00777 | NP_650428 | NP_648106<br>AAL28680 | NP_610719 | XP_002610333 | NP_523935 | NP_476678 |
| | | ***Lottia gigantea*** | Acc Num NA | Acc Num NA | Acc Num NA | Acc Num NA | Acc Num NA | XP_394668 | Acc Num NA | Acc Num NA | Acc Num NA | Acc Num NA | |
| | | ***Apis mellifera*** | XP_395059 | XP_393700 | | XP_001123117 | XP_001120878 | XP_394668 | XP_624624 | XP_623860 | XP_395593 | | |
| | | ***Caenorhabditis elegans*** | NP_001040658 | NP_499453 | NP_509262 | NP_506193 | NP_493687 | NP_492228 | NP_498625 | NP_497865 | NP_500566 | NP_491160 | |
| | | ***Brugia malayi*** | XP_001894059 | XP_001899737 | XP_001895586 | XP_001901686 | EDP30362 | XP_001892554 | XP_001897363 | XP_001895635 | XP_001892742 | XP_001894870 | |
| | | ***Nematostella vectensis*** | XP_001641659 | XP_001626236 | Acc Num NA | Acc Num NA | Acc Num NA | XP_001624621 | XP_001639589 | XP_001641695 | XP_001626696 | XP_001635666 | |
| | | ***Helobdella robusta*** | Acc Num NA | Acc Num NA | Acc Num NA | Acc Num NA | Acc Num NA | Acc Num NA | Acc Num NA | Acc Num NA | Acc Num NA | Acc Num NA | |
| | | ***Daphnia pulex*** | Acc Num NA | Acc Num NA | Acc Num NA | Acc Num NA | Acc Num NA | Acc Num NA | Acc Num NA | Acc Num NA | Acc Num NA | Acc Num NA | |
| | **Capsaspora** | ***Capsaspora owczarzaki*** | Acc Num NA | Acc Num NA | Acc Num NA | Acc Num NA | Acc Num NA | Acc Num NA | Acc Num NA | Acc Num NA | Acc Num NA | Acc Num NA | |
| | **Fungi** | ***Cryptococcus neoformans*** | XP_568851 | XP_775936 | XP_570356 | XP_567136 | XP_566838 | | XP_569515 | XP_567466 | XP_778346 | XP_569001 | |
| | | ***Ustilago maydis*** | XP_759390 | XP_760536 | XP_758738 | XP_758200 | XP_761813 | | | XP_759887 | XP_760279 | XP_759409 | |
| | | ***Aspergillus fumigatus*** | EDP52912 | XP_755741 | EDP54236 | EDP55114 | XP_749676 | XP_747613 | XP_755006 | EDP55189 | EAL92045 | XP_753668 | |
| | | ***Schizosaccharomyces pombe*** | NP_596049 | NP_593260 | NP_588151 | XP_001713063 | NP_592917 | NP_593137 | NP_596778 | NP_593535 | NP_595195 | NP_587903 | NP_010398 |
| | | ***Saccharomyces cerevisiae*** | NP_116647 | NP_012461 | NP_010281 | NP_012238 | NP_010466 | EDN63124 | NP_116683 | NP_013793 | NP_010297 | EDN61688 | NP8595904 |
| | | ***Neurospora crassa*** | XP_961409 | XP_957879 | XP_964217 | XP_960650 | XP_961787 | XP_961389 | | XP_956688 | XP_955967 | XP_956590 | |
| | | ***Encephalitozoon cuniculi*** | NP_584776 | XP_955745 | | NP_584770 | | | | | | NP_585963 | |
| | | ***Enterocytozoon bieneusi H348*** | XP_002649733 | | | | | | | | | XP_001827760 | |
| | | ***Nosema ceranae BRL01*** | | XP_002995833 | | XP_002996063 | | | | | | XP_002996251 | |
| | | ***Encephalitozoon intestinalis ATCC 50506*** | XP_003072739 | XP_003073682 | | XP_003072732 | | | | | | XP_003073153 | |
| | | ***Batrachochytrium dendrobatidis*** | Acc Num NA | Acc Num NA | Acc Num NA | Acc Num NA | Acc Num NA | | Acc Num NA | Acc Num NA | Acc Num NA | Acc Num NA | |
| | | ***Spizellomyces punctatus*** | Acc Num NA | Acc Num NA | Acc Num NA | Acc Num NA | Acc Num NA | Acc Num NA | Acc Num NA | Acc Num NA | Acc Num NA | Acc Num NA | |
| **Apusozoa** | | ***Thecamonas trahens*** | Acc Num NA | Acc Num NA | Acc Num NA | Acc Num NA | Acc Num NA | | Acc Num NA | | | Acc Num NA | |
| **Amoebozoa** | | ***Dictyostelium discoideum*** | XP_629977 | XP_643274 | XP_642955 | XP_638963 | XP_639229 | | XP_641425 | XP_643624 | | XP_635802 | |
| | | ***Entamoeba histolytica*** | XP_656581 | XP_655216 | XP_657283 | | | | | | | XP_651118 | |
| **Excavata** | **Metamonada** | ***Giardia intestinalis*** | EET00911 | EET02057 | | | | | | | | CAD67965 | |
| | | ***Trichomonas vaginalis*** | XP_001329543 | XP_001319807<br>XP_001328416 | XP_001329298 | ABC61972 | XP_001581890 | | | XP_001329031 | | XP_001582914 | |
| | **Euglenozoa** | ***Leishmania major*** | XP_843456 | XP_001681030 | XP_001687586 | XP_001686731 | | | XP_001682791 | XP_001681555 | | XP_001682914 | |
| | | ***Leishmania infantum*** | XP_001469160 | XP_001463342 | XP_001463072 | XP_001469747 | | | XP_001465213 | XP_001463886 | | XP_001465288 | |
| | | ***Trypanosoma cruzi*** | XP_817950 | XP_815055 | XP_819913 | XP_809775 | | | XP_818808 | XP_821239 | | XP_809313 | |
| | | ***Trypanosoma brucei*** | XP_827441 | XP_845021 | XP_846310 | XP_822844 | | | XP_001218868 | XP_828716 | | CBH09022 | |
| | **Heterolobosoa** | ***Naegleria gruberi*** | XP_002682487 | XP_002670278 | XP_002679151 | XP_002673603 | XP_002683017 | | XP_002680835 | XP_002671795 | | Acc Num NA | |
| **Alveolata** | **Ciliata** | ***Tetrahymena thermophila*** | XP_001025504 | XP_001008623 | | | | | | | | XP_001013249 | |
| | | ***Paramecium tetraurelia*** | XP_001426830 | XP_001443869 | | | | | | | | XP_001458342 | |
| | | ***Oxytricha trifallax*** | ? | ? | ? | | | | | | | | |
| | **Apicomplexa** | ***Plasmodium yoelii*** | XP_726100 | XP_725208 | | XP_730250 | | | | | | XP_726982 | |
| | | ***Plasmodium falciparum*** | XP_001347988 | XP_001351448 | | XP_001348712 | | | | | | XP_002808832 | |
| | | ***Cryptosporidium hominis*** | XP_666991 | XP_665748 | XP_668359 | | | | | | | XP_665802 | |
| | | ***Babesia bovis*** | XP_001610390 | XP_001611424 | | XP_001609890 | | | | | | Acc Num NA | |
| | | ***Theileria annulata*** | XP_954442 | | | XP_952886 | | | | | | | |
| | | ***Toxoplasma gondii*** | XP_002368310 | AAZ57430 | | | | | | | | EEE32077 | |
| **Heterokonta** | **Blastocystae** | ***Blastocystis hominis*** | CBK21095 | CBK23979 | | | | | | | | CBK21518 | |
| | **Phaeophyceae** | ***Ectocarpus siliculosus*** | CBN77803 | CBN79764 | CBJ33525 | CBJ33097 | CBJ31249 | | CBJ31471 | CBJ25477 | | CBJ25668 | |
| | **Oomycota** | ***Phytophthora ramorum*** | Acc Num NA | Acc Num NA | Acc Num NA | Acc Num NA | Acc Num NA | | Acc Num NA | Acc Num NA | | | |
| | | ***Phytophthora infestans*** | XP_002998013 | XP_002997684 | XP_002902993 | XP_002904221 | XP_002906797 | | XP_002902720 | XP_002996972 | | XP_002906565 | |
| | **Bacillariophyta** | ***Thalassiosira pseudonana*** | XP_002296149 | | XP_002292911 | | XP_002287148 | | XP_002287134 | XP_002291327 | | XP_002286533 | |
| | | ***Phaeodactylum tricornutum*** | XP_002177808 | XP_002184568 | XP_002178341 | | XP_002185061 | | XP_002177583 | | | XP_002182854 | |
| | | ***Aureococcus anophagefferens*** | Acc Num NA | Acc Num NA | Acc Num NA | Acc Num NA | Acc Num NA | | | | | Acc Num NA | |
| **Plantae** | **Viridiplantae** | ***Oryza sativa*** | NP_001067404 | NP_001045803 | NP_001053148 | NP_001054841 | EEE66449 | CAD40351 | NP_001055381 | EEE55732 | NP_001064956 | EEC74078 | |
| | | ***Arabidopsis thaliana*** | NP_191027 | NP_001077968 | NP_568586 | NP_566119 | NP_197058 | BAF00935 | NP_194868 | NP_177883<br>NP_001119390 | NP_176298 | NP_194028 | |
| | | ***Chlamydomonas reinhardtii*** | XP_001700607 | XP_001698278 | XP_001696730 | XP_001699607 | XP_001697971 | | | XP_001690069 | | XP_001689575 | |
| | | ***Ostreococcus tauri*** | XP_003078284 | XP_003081202 | XP_003079934 | XP_003074534 | XP_003075000 | XP_003082234 | XP_003081729 | XP_003074597 | XP_003081951 | XP_003074856 | |
| | | ***Ostreococcus lucimarinus*** | XP_001416713 | XP_001419553 | XP_001418499 | XP_001415613 | XP_001416473 | XP_001420595 | XP_001420193 | XP_001415641 | XP_001420300 | XP_001416421 | |
| | | ***Physcomitrella patens*** | XP_001767264 | XP_001784554 | XP_001765550 | XP_001776000 | | XP_001773150 | XP_001763476 | XP_001778595 | XP_001781920 | XP_001759110 | |
| | | ***Chlorella vulgaris*** | Acc Num NA | Acc Num NA | Acc Num NA | Acc Num NA | Acc Num NA | | Acc Num NA | Acc Num NA | Acc Num NA | Acc Num NA | |
| | | ***Micromonas pusilla*** | XP_003055298 | XP_003056807 | XP_003062115 | XP_003056469 | XP_003056333 | XP_003055504 | XP_003063893 | XP_003062863 | | | |
| | | ***Selaginella moellendorffii*** | XP_002990356 | Acc Num NA | XP_002985553 | XP_002966356 | XP_002981324 | Acc Num NA | XP_002960198 | XP_002990499 | XP_002977658 | XP_002975469 | |
| | **Rhodophyta** | ***Cyanidioschyzon merolae*** | Acc Num NA | Acc Num NA | CML311C | CMO331C | | | CMT193C | | | Acc Num NA | |
| | | ***Galdieria sulphuraria*** | Acc Num NA | Acc Num NA | ? | Acc Num NA | ? | | Acc Num NA | Acc Num NA | | Acc Num NA | |
| **Haptophyta** | | ***Emiliania huxleyi*** | Acc Num NA | ? | Acc Num NA | Acc Num NA | Acc Num NA | | ? | Acc Num NA | | Acc Num NA | |
