# Supplementary material for: The phylogenomic analysis of the anaphase promoting complex and its targets points to complex and modern-like control of the cell cycle in the last common ancestor of eukaryotes
Source: BMC Evol Biol. 2011 Sep 23;11:265. doi: 10.1186/1471-2148-11-265 (PMC3195147; doi:10.1186/1471-2148-11-265)
Supplement: Additional file 4 — Figure S1. Bayesian phylogenetic tree of the adaptor/co-activator Cdh1 inferred with Mrbayes. Numbers at nodes correspond to posterior probabilities (PP) estimated by MrBayes and bootstrap values (BV) estimated by TreeFinder (only PP and BV greater than 0.5 and 50%, respectively, are shown). The scale bar indicates the average number of substitutions per site. [file 1471-2148-11-265-S4.PDF]

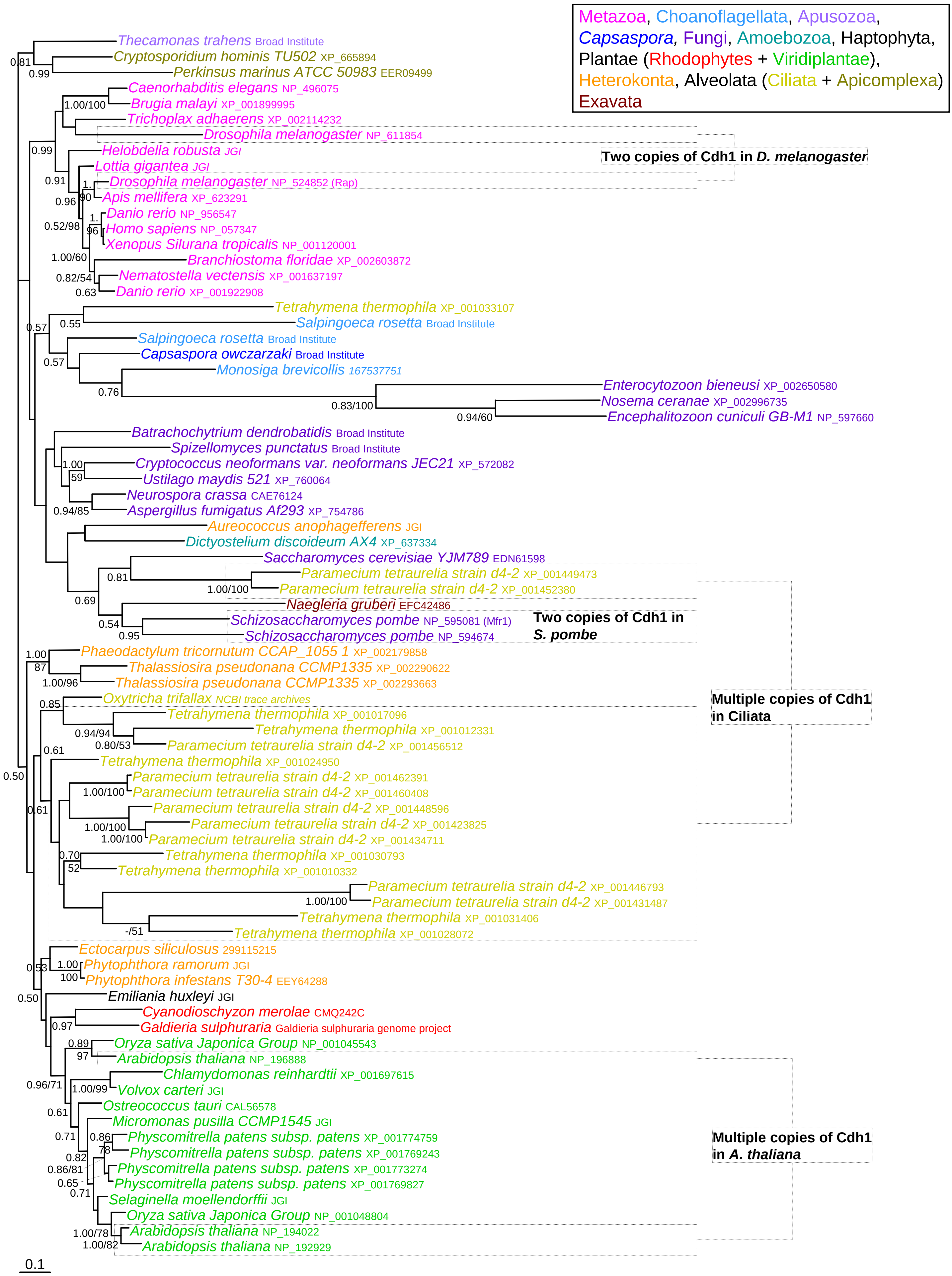

Metazoa, Choanoflagellata, Apusozoa, Capsaspora, Fungi, Amoebozoa, Haptophyta, Plantae (Rhodophytes + Viridiplantae), Heterokonta, Alveolata (Ciliata + Apicomplexa) Exavata
Thecamonas trahens Broad Institute
Cryptosporidium hominis TU502 XP_665894
Perkinsus marinus ATCC 50983 EER09499
Caenorhabditis elegans NP_496075
Brugia malayi XP_001899995
Trichoplax adhaerens XP_002114232
Drosophila melanogaster NP_611854
Helobdella robusta JGI
Lottia gigantea JGI
Drosophila melanogaster NP_524852 (Rap)
Apis mellifera XP_623291
Danio rerio NP_956547
Homo sapiens NP_057347
Xenopus Silurana tropicalis NP_001120001
Branchiostoma floridae XP_002603872
Nematostella vectensis XP_001637197
Danio rerio XP_001922908
Two copies of Cdh1 in D. melanogaster
Tetrahymena thermophila XP_001033107
Salpingoeca rosetta Broad Institute
Salpingoeca rosetta Broad Institute
Capsaspora owczarzaki Broad Institute
Monosiga brevicollis 167537751
Enterocytozoon bieneusi XP_002650580
Nosema ceranae XP_002996735
Encephalitozoon cuniculi GB-M1 NP_597660
Batrachochytrium dendrobatidis Broad Institute
Spizellomyces punctatus Broad Institute
Cryptococcus neoformans var. neoformans JEC21 XP_572082
Ustilago maydis 521 XP_760064
Neurospora crassa CAE76124
Aspergillus fumigatus Af293 XP_754786
Aureococcus anophagefferens JGI
Dictyostelium discoideum AX4 XP_637334
Saccharomyces cerevisiae YJM789 EDN61598
Paramecium tetraurelia strain d4-2 XP_001449473
Paramecium tetraurelia strain d4-2 XP_001452380
Naegleria gruberi EFC42486
Schizosaccharomyces pombe NP_595081 (Mfr1)
Schizosaccharomyces pombe NP_594674
Two copies of Cdh1 in S. pombe
Phaeodactylum tricornutum CCAP_1055 1 XP_002179858
Thalassiosira pseudonana CCMP1335 XP_002290622
Thalassiosira pseudonana CCMP1335 XP_002293663
Oxytricha trifallax NCBI trace archives
Tetrahymena thermophila XP_001017096
Tetrahymena thermophila XP_001012331
Paramecium tetraurelia strain d4-2 XP_001456512
Tetrahymena thermophila XP_001024950
Paramecium tetraurelia strain d4-2 XP_001462391
Paramecium tetraurelia strain d4-2 XP_001460408
Paramecium tetraurelia strain d4-2 XP_001448596
Paramecium tetraurelia strain d4-2 XP_001423825
Paramecium tetraurelia strain d4-2 XP_001434711
Tetrahymena thermophila XP_001030793
Tetrahymena thermophila XP_001010332
Paramecium tetraurelia strain d4-2 XP_001446793
Paramecium tetraurelia strain d4-2 XP_001431487
Tetrahymena thermophila XP_001031406
Tetrahymena thermophila XP_001028072
Multiple copies of Cdh1 in Ciliata
Ectocarpus siliculosus 299115215
Phytophthora ramorum JGI
Phytophthora infestans T30-4 EEY64288
Emiliania huxleyi JGI
Cyanodioschyzon merolae CMQ242C
Galdieria sulphuraria Galdieria sulphuraria genome project
Oryza sativa Japonica Group NP_001045543
Arabidopsis thaliana NP_196888
Chlamydomonas reinhardtii XP_001697615
Volvox carteri JGI
Ostreococcus tauri CAL56578
Micromonas pusilla CCMP1545 JGI
Physcomitrella patens subsp. patens XP_001774759
Physcomitrella patens subsp. patens XP_001769243
Physcomitrella patens subsp. patens XP_001773274
Physcomitrella patens subsp. patens XP_001769827
Selaginella moellendorffii JGI
Oryza sativa Japonica Group NP_001048804
Arabidopsis thaliana NP_194022
Arabidopsis thaliana NP_192929
Multiple copies of Cdh1 in A. thaliana
0.1
