# Supplementary material for: The phylogenomic analysis of the anaphase promoting complex and its targets points to complex and modern-like control of the cell cycle in the last common ancestor of eukaryotes
Source: BMC Evol Biol. 2011 Sep 23;11:265. doi: 10.1186/1471-2148-11-265 (PMC3195147; doi:10.1186/1471-2148-11-265)
Supplement: Additional file 5 — Figure S2. Bayesian phylogenetic tree of the adaptor/co-activator Cdc20 inferred with Mrbayes. Numbers at nodes correspond to posterior probabilities (PP) estimated by MrBayes and bootstrap values (BV) estimated by TreeFinder (only PP and BV greater than 0.5 and 50%, respectively, are shown). The scale bar indicates the average number of substitutions per site. [file 1471-2148-11-265-S5.PDF]

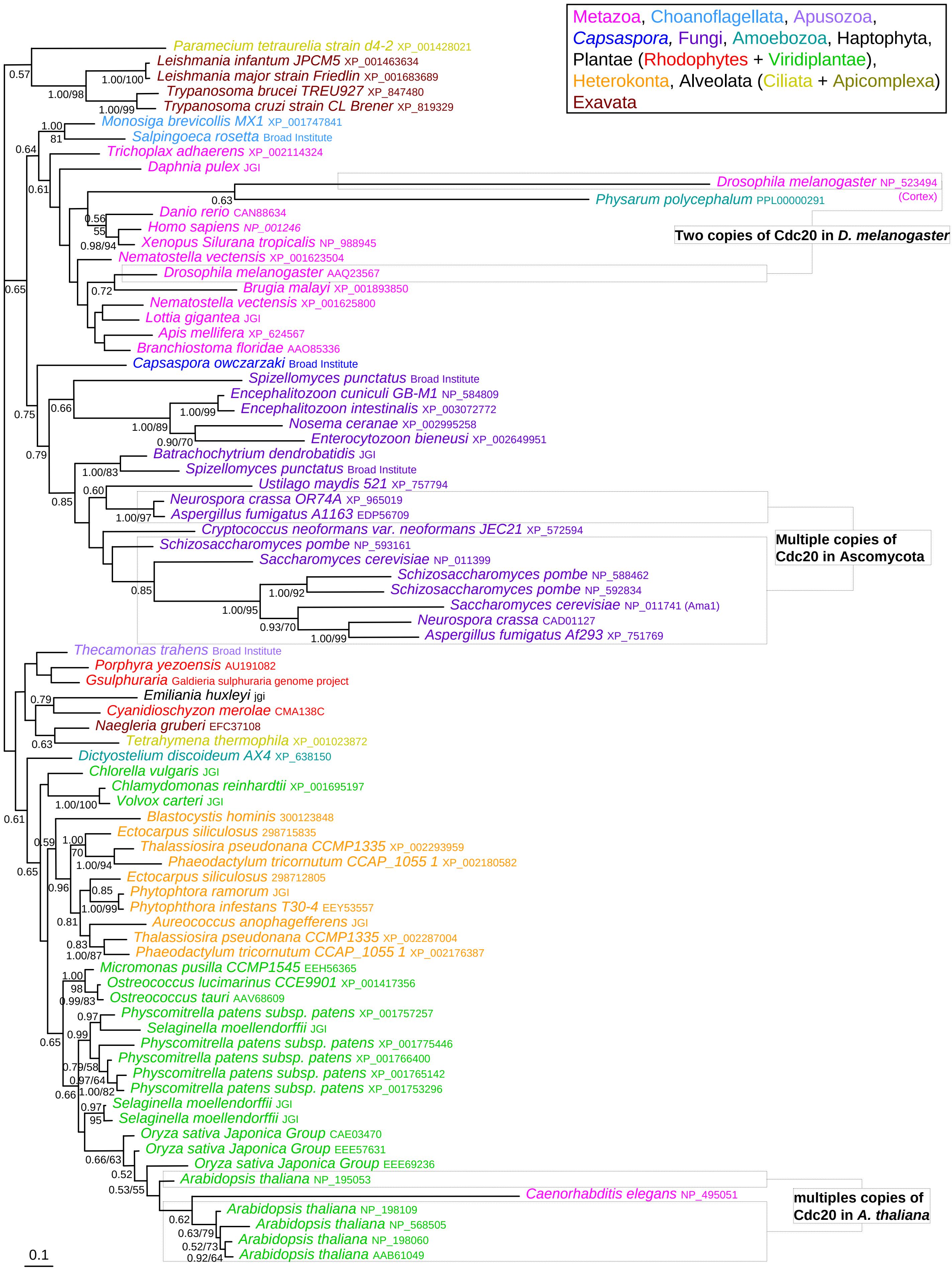

Metazoa, Choanoflagellata, Apusozoa, Capsaspora, Fungi, Amoebozoa, Haptophyta, Plantae (Rhodophytes + Viridiplantae), Heterokonta, Alveolata (Ciliata + Apicomplexa) Exavata
Paramecium tetraurelia strain d4-2 XP_001428021
Leishmania infantum JPCM5 XP_001463634
Leishmania major strain Friedlin XP_001683689
Trypanosoma brucei TREU927 XP_847480
Trypanosoma cruzi strain CL Brener XP_819329
Monosiga brevicollis MX1 XP_001747841
Salpingoeca rosetta Broad Institute
Trichoplax adhaerens XP_002114324
Daphnia pulex JGI
Drosophila melanogaster NP_523494 (Cortex)
Physarum polycephalum PPL00000291
Danio rerio CAN88634
Homo sapiens NP_001246
Xenopus Silurana tropicalis NP_988945
Two copies of Cdc20 in D. melanogaster
Nematostella vectensis XP_001623504
Drosophila melanogaster AAQ23567
Brugia malayi XP_001893850
Nematostella vectensis XP_001625800
Lottia gigantea JGI
Apis mellifera XP_624567
Branchiostoma floridae AAO85336
Capsaspora owczarzaki Broad Institute
Spizellomyces punctatus Broad Institute
Encephalitozoon cuniculi GB-M1 NP_584809
Encephalitozoon intestinalis XP_003072772
Nosema ceranae XP_002995258
Enterocytozoon bieneusi XP_002649951
Batrachochytrium dendrobatidis JGI
Spizellomyces punctatus Broad Institute
Ustilago maydis 521 XP_757794
Neurospora crassa OR74A XP_965019
Aspergillus fumigatus A1163 EDP56709
Cryptococcus neoformans var. neoformans JEC21 XP_572594
Schizosaccharomyces pombe NP_593161
Saccharomyces cerevisiae NP_011399
Multiple copies of Cdc20 in Ascomycota
Schizosaccharomyces pombe NP_588462
Schizosaccharomyces pombe NP_592834
Saccharomyces cerevisiae NP_011741 (Ama1)
Neurospora crassa CAD01127
Aspergillus fumigatus Af293 XP_751769
Thecamonas trahens Broad Institute
Porphyra yezoensis AU191082
Gsulphuraria Galdieria sulphuraria genome project
Emiliania huxleyi jgi
Cyanidioschyzon merolae CMA138C
Naegleria gruberi EFC37108
Tetrahymena thermophila XP_001023872
Dictyostelium discoideum AX4 XP_638150
Chlorella vulgaris JGI
Chlamydomonas reinhardtii XP_001695197
Volvox carteri JGI
Blastocystis hominis 300123848
Ectocarpus siliculosus 298715835
Thalassiosira pseudonana CCMP1335 XP_002293959
Phaeodactylum tricornutum CCAP_1055 1 XP_002180582
Ectocarpus siliculosus 298712805
Phytophtora ramorum JGI
Phytophthora infestans T30-4 EEY53557
Aureococcus anophagefferens JGI
Thalassiosira pseudonana CCMP1335 XP_002287004
Phaeodactylum tricornutum CCAP_1055 1 XP_002176387
Micromonas pusilla CCMP1545 EEH56365
Ostreococcus lucimarinus CCE9901 XP_001417356
Ostreococcus tauri AAV68609
Physcomitrella patens subsp. patens XP_001757257
Selaginella moellendorffii JGI
Physcomitrella patens subsp. patens XP_001775446
Physcomitrella patens subsp. patens XP_001766400
Physcomitrella patens subsp. patens XP_001765142
Physcomitrella patens subsp. patens XP_001753296
Selaginella moellendorffii JGI
Selaginella moellendorffii JGI
Oryza sativa Japonica Group CAE03470
Oryza sativa Japonica Group EEE57631
Oryza sativa Japonica Group EEE69236
Arabidopsis thaliana NP_195053
Caenorhabditis elegans NP_495051
multiples copies of Cdc20 in A. thaliana
Arabidopsis thaliana NP_198109
Arabidopsis thaliana NP_568505
Arabidopsis thaliana NP_198060
Arabidopsis thaliana AAB61049
0.1
