# Supplementary material for: The phylogenomic analysis of the anaphase promoting complex and its targets points to complex and modern-like control of the cell cycle in the last common ancestor of eukaryotes
Source: BMC Evol Biol. 2011 Sep 23;11:265. doi: 10.1186/1471-2148-11-265 (PMC3195147; doi:10.1186/1471-2148-11-265)
Supplement: Additional file 6 — Table S3. Table showing the conserved functional domains present in homologues of APC/C subunits and activators. [file 1471-2148-11-265-S6.PDF]

Supplementary Table S3. Domain composition of homologues of APC/C subunits and adaptors/co-activators. Domain presence was determined according to the Pfam database. A "-" indicates the absence of conserved domain in the corresponding protein. Empty cells indicate that no homologue of the protein has been detected in the corresponding genome. Profile accession numbers: APC10 (PF03256.7), zf-C3HC4 (PF00097.15), APC2 (PF08672.1), Cullin (PF00888.12), PC_rep (PF01851.12), APC8 (PF04049.4) and WD40 (PF00400.22). Profiles corresponding to TPR domains are either TPR_1 (PF00515.18), TPR_2 (PF07719.7) or TPR_4 (PF07721.5).

			**Core subunits**																**Co-activators**						
			**Catalytic arm**			**Structural complex**			**TPR arm and associated proteins**									**Unknown**	**Mitosis co-activators**			**Meiotic co-activator**			
			**Apc10**	**Apc11**	**Apc2**	**Apc1**	**Apc4**	**Apc5**	**Apc8**	**Apc6**	**Apc3**	**Apc7**	**Apc12**	**Apc13**	**Apc16**	**Apc9**	**Apc15**	**Apc14**	**Cdc20**	**Cdh1**	**uncertain**	**Mfr1**	**Ama1**	**Rap**	**Cortex**
**Opisthokonta**	**Choanoflagellata**	***Monosiga brevicollis***	APC10		APC2/Cullin	PC_rep	-		APC8/TPR	TPR	TPR	TPR							WD40	WD40					
		Salpingoeca rosetta	APC10		Cullin	PC_rep			APC8/TPR	TPR	TPR								WD40	WD40					
	**Metazoa**	***Trichoplax adhaerens***	APC10	zf-C3HC4	APC2/Cullin	PC_rep	-	TPR	APC8/TPR	TPR	TPR	TPR		-					WD40	WD40					
		Homo sapiens	APC10	zf-C3HC4	APC2/Cullin	PC_rep	-	TPR	APC8/TPR	TPR	TPR	TPR	-	-	-				WD40	WD40					
		Danio rerio	APC10	zf-C3HC4	APC2/Cullin	PC_rep	-	TPR	APC8/TPR	TPR	TPR	TPR	-	-	-				WD40	WD40					
		Branchiostoma floridae	APC10	zf-C3HC4	APC2/Cullin	PC_rep	-	TPR	APC8/TPR	TPR	TPR	TPR	-	-					WD40	WD40					
		Drosophila melanogaster	APC10	zf-C3HC4	APC2/Cullin	PC_rep	-	TPR	APC8/TPR	TPR	TPR	TPR	-	-					WD40	WD40				WD40	WD40
		Lottia gigantea	APC10	zf-C3HC4	APC2/Cullin	PC_rep	-	TPR	APC8/TPR	TPR	TPR	TPR	-	-					WD40	WD40					
		Apis mellifera	APC10	zf-C3HC4	APC2/Cullin	PC_rep	-	-	APC8/TPR	TPR	TPR	TPR	-	-					WD40	WD40					
		Caenorhabditis elegans	APC10	zf-C3HC4	APC2/Cullin	PC_rep	-	-	APC8/TPR	TPR	TPR	TPR							WD40	WD40					
		Brugia malayi	APC10	zf-C3HC4	APC2/Cullin	PC_rep	-	TPR	APC8/TPR	TPR	TPR	TPR	-	-					WD40	WD40					
		Nematostella vectensis	APC10	zf-C3HC4	APC2/Cullin	PC_rep	-	TPR	APC8/TPR	TPR	TPR	TPR							WD40	WD40					
		Helobdella robusta	APC10	zf-C3HC4	APC2/Cullin	PC_rep	-	-	APC8/TPR	TPR	TPR	TPR		-						WD40					
		Daphnia pulex	APC10	zf-C3HC4	APC2/Cullin	PC_rep	-	-	APC8/TPR	TPR	TPR	TPR		-					WD40	WD40					
	**Capsaspora**	***Capsaspora owczarzaki***	APC10	zf-C3HC4	APC2/Cullin	PC_rep	-	TPR	APC8/TPR		TPR	TPR							WD40	WD40					
	**Fungi**	***Cryptococcus neoformans***	APC10	zf-C3HC4	-	PC_rep	-	-	APC8/TPR	TPR	TPR								WD40	WD40					
		Ustilago maydis	APC10	zf-C3HC4	-	PC_rep	-	-	APC8/TPR	TPR	TPR								WD40	WD40					
		Aspergillus fumigatus	APC10	zf-C3HC4	APC2/Cullin	PC_rep	-	-	APC8/TPR	TPR	TPR		-	-					WD40	WD40			WD40		
		Schizosaccharomyces pombe	APC10	zf-C3HC4	APC2/Cullin	PC_rep	-	-	APC8/TPR	TPR	TPR		-	-			Apc15p	-	WD40	WD40		WD40	WD40		
		Saccharomyces cerevisiae	APC10	zf-C3HC4	APC2/Cullin	PC_rep	-	-	APC8/TPR	TPR	TPR		-	-		-	Apc15p		WD40	WD40			WD40		
		Neurospora crassa	APC10	zf-C3HC4	APC2/Cullin	PC_rep	-	-	APC8/TPR	TPR	TPR		-	-					WD40	WD40			WD40		
		Encephalitozoon cuniculi	APC10			PC_rep			TPR	TPR	TPR								WD40		WD40				
		Enterocytozoon bieneusi H348	APC10	zf-C3HC4		PC_rep				TPR	TPR								WD40	WD40					
		Nosema ceranae BRL01	APC10			PC_rep			TPR	TPR	TPR								WD40	WD40					
		Encephalitozoon intestinalis ATCC 50506	APC10			PC_rep			TPR	TPR	TPR								WD40		WD40				
		Batrachochytrium dendrobatidis	APC10	zf-C3HC4	Cullin	PC_rep		-	APC8/TPR	TPR	TPR	TPR							WD40	WD40					
		Spizellomyces punctatus		zf-C3HC4	APC2/Cullin	PC_rep	-		APC8/TPR	TPR	TPR	TPR							WD40	WD40					
**Apusozoa**		***Thecamonas trahens***	APC10	zf-C3HC4	APC2/Cullin	PC_rep	-	TPR	APC8/TPR	TPR	TPR								WD40	WD40					
**Amoebozoa**		***Dictyostelium discoideum***	APC10	zf-C3HC4	APC2/Cullin	PC_rep	-	-	APC8/TPR	TPR	TPR	TPR	-	-					WD40	WD40					
		Entamoeba histolytica	APC10	zf-C3HC4		PC_rep															WD40				
**Excavata**	**Metamonada**	***Giardia intestinalis***		zf-C3HC4																					
		Trichomonas vaginalis	APC10	zf-C3HC4	Cullin	PC_rep			APC8/TPR	TPR	TPR	TPR									WD40				
	**Euglenozoa**	***Leishmania major***	APC10	zf-C3HC4	Cullin	PC_rep			TPR	TPR	TPR								WD40						
		Leishmania infantum	APC10	zf-C3HC4	Cullin	PC_rep			TPR	TPR	TPR								WD40						
		Trypanosoma cruzi	APC10	zf-C3HC4	Cullin	PC_rep			TPR	TPR	TPR								WD40						
		Trypanosoma brucei	APC10	zf-C3HC4	APC2/Cullin	PC_rep			TPR	TPR	TPR								WD40						
	**Heterolobosoa**	***Naegleria gruberi***	APC10		APC2/Cullin	PC_rep			TPR	TPR	TPR								WD40	WD40					
**Alveolata**	**Ciliata**	***Tetrahymena thermophila***	APC10	zf-C3HC4	APC2/Cullin	PC_rep	-		APC8/TPR	TPR	TPR								WD40	WD40					
		Paramecium tetraurelia	APC10	zf-C3HC4	Cullin	PC_rep	-		APC8/TPR	TPR	TPR								WD40	WD40					
		Oxytricha trifallax	APC10	zf-C3HC4	APC2/Cullin	PC_rep			TPR	TPR	TPR									WD40					
	**Apicomplexa**	***Plasmodium yoelii***	APC10	zf-C3HC4							TPR										WD40				
		Plasmodium falciparum	APC10	zf-C3HC4							TPR										WD40				
		Cryptosporidium hominis		zf-C3HC4		PC_rep			APC8/TPR	TPR	TPR									WD40					
		Babesia bovis																							
		Theileria annulata																							
		Toxoplasma gondii	APC10	zf-C3HC4		PC_rep																			
**Heterokonta**	**Blastocystae**	***Blastocystis hominis***	APC10						TPR	TPR									WD40						
	**Phaeophyceae**	***Ectocarpus siliculosus***	APC10	zf-C3HC4	APC2/Cullin	PC_rep	-	-	TPR	TPR	TPR	TPR							WD40	WD40					
	**Oomycota**	***Phytophthora ramorum***	APC10	zf-C3HC4	Cullin			-	APC8/TPR	TPR	TPR	TPR	-						WD40	WD40					
		Phytophthora infestans	APC10		APC2/Cullin	PC_rep	-	TPR	APC8/TPR	TPR	TPR	TPR	-	-					WD40	WD40					
	**Bacillariophyta**	***Thalassiosira pseudonana***	APC10	zf-C3HC4	APC2/Cullin	PC_rep		TPR	TPR		TPR	TPR		-					WD40	WD40					
		Phaeodactylum tricornutum	APC10	zf-C3HC4	APC2/Cullin				APC8/TPR	TPR	TPR	TPR	-	-					WD40	WD40					
		Aureococcus anophagefferens	APC10		APC2/Cullin			-	APC8/TPR	TPR	TPR	TPR							WD40	WD40					
**Plantae**	**Viridiplantae**	***Oryza sativa***	APC10	zf-C3HC4	APC2/Cullin	PC_rep	-	TPR	APC8/TPR	TPR	TPR	TPR		-					WD40	WD40					
		Arabidopsis thaliana	APC10	zf-C3HC4	APC2/Cullin	PC_rep	-	TPR	APC8/TPR	TPR	TPR	TPR	-	-					WD40	WD40					
		Chlamydomonas reinhardtii	APC10	zf-C3HC4	Cullin	PC_rep	-		APC8/TPR	TPR	TPR			-					WD40	WD40					
		Ostreococcus tauri	APC10	zf-C3HC4	APC2/Cullin	PC_rep	-	-	APC8/TPR	TPR	TPR	TPR							WD40	WD40					
		Ostreococcus lucimarinus	APC10	zf-C3HC4	APC2/Cullin	PC_rep	-	-	TPR	TPR	TPR			-					WD40						
		Physcomitrella patens	APC10	zf-C3HC4	APC2/Cullin	PC_rep	-	-	APC8/TPR	TPR	TPR	TPR		-					WD40	WD40					
		Chlorella vulgaris	APC10	zf-C3HC4	APC2/Cullin	PC_rep	-	TPR	APC8/TPR	TPR	TPR								WD40	WD40					
		Micromonas pusilla	APC10	zf-C3HC4	APC2/Cullin	PC_rep	-	TPR	APC8/TPR	TPR	TPR	TPR		-					WD40	WD40					
		Selaginella moellendorffii	APC10	zf-C3HC4	APC2/Cullin	PC_rep	-	TPR	APC8/TPR	TPR	TPR	TPR							WD40	WD40					
	**Rhodophyta**	***Cyanidioschyzon merolae***	APC10	zf-C3HC4	APC2/Cullin	PC_rep			APC8/TPR	TPR	TPR								WD40	WD40					
		Galdieria sulphuraria	APC10	zf-C3HC4	APC2/Cullin	PC_rep			TPR	TPR	TPR								WD40	WD40					
**Haptophyta**		***Emiliania huxleyi***	APC10	zf-C3HC4	APC2/Cullin	PC_rep	-		APC8/TPR	TPR	TPR								WD40	WD40					
