# Supplementary material for: The phylogenomic analysis of the anaphase promoting complex and its targets points to complex and modern-like control of the cell cycle in the last common ancestor of eukaryotes
Source: BMC Evol Biol. 2011 Sep 23;11:265. doi: 10.1186/1471-2148-11-265 (PMC3195147; doi:10.1186/1471-2148-11-265)
Supplement: Additional file 7 — Table S4. Table showing the conserved functional domains present in homologues of APC/C main targets. [file 1471-2148-11-265-S7.PDF]

Supplementary Table S4. Domain composition of homologues of APC/C main targets. Domain presence was determined according to the Pfam database. A "-" indicates the absence of conserved domain in the corresponding protein. Empty cells indicate that no homologue of the protein has been detected in the corresponding genome. Profile accession numbers: SMC_hinge (PF06470.4), SMC_N (PF02463.9), Rad21_Rec8 (PF04824.7), Rad21_Rec8_N (PF04825.4), HEAT (PF02985.12), STAG (PF08514.2), WAPL (PF07814.3), Peptidase_C50 (PF03568.7) and Securin (PF04856.4).

			Core complex				Loading complex		Cohesion establishment	Maintenance		Dissolution	
			**Smc1**	**Smc3**	**Scc1**	**Scc3**	**Scc2**	**Scc4**	**Eco1**	**Pds5**	**Wpl1**	**Separase**	**Securin**
**Opisthokonta**	**Choanoflagellata**	***Monosiga brevicollis***	-	SMC_hinge/SMC_N	Rad21_Rec8/Rad21_Rec8_N	-	HEAT			HEAT		Peptidase_C50	
		Salpingoeca rosetta	SMC_hinge/SMC_N	-	Rad21_Rec8/Rad21_Rec8_N	NA	HEAT		-	HEAT	WAPL	Peptidase_C50	
	**Metazoa**	***Trichoplax adhaerens***	-	-	Rad21_Rec8/Rad21_Rec8_N	HEAT/STAG	HEAT	TPR	-	HEAT	WAPL	-	
		Homo sapiens	-	-	Rad21_Rec8/Rad21_Rec8_N	HEAT	HEAT	TPR	-	HEAT	WAPL	Peptidase_C50	Securin
		Danio rerio	-	-	Rad21_Rec8/Rad21_Rec8_N	HEAT	HEAT	-	-	-	WAPL	Peptidase_C50	Securin
		Branchiostoma floridae			Rad21_Rec8_N	STAG	HEAT	-	-	HEAT	WAPL	Peptidase_C50	
		Drosophila melanogaster	-	-	Rad21_Rec8/Rad21_Rec8_N	HEAT	HEAT	TPR	-	-	WAPL	Peptidase_C50	-
		Lottia gigantea	-	SMC_N	Rad21_Rec8/Rad21_Rec8_N	HEAT	HEAT	-	-	HEAT	WAPL	Peptidase_C50	
		Apis mellifera	SMC_hinge/SMC_N	SMC_hinge/SMC_N		HEAT	HEAT	TPR	-	HEAT	WAPL		
		Caenorhabditis elegans	SMC_hinge/SMC_N	SMC_hinge/SMC_N	Rad21_Rec8/Rad21_Rec8_N	STAG	HEAT	TPR	-	HEAT	WAPL	Peptidase_C50	-
		Brugia malayi	SMC_hinge/SMC_N	SMC_hinge/SMC_N	Rad21_Rec8/Rad21_Rec8_N	HEAT	HEAT	-	-	HEAT	WAPL	Peptidase_C50	
		Nematostella vectensis	-	-	Rad21_Rec8/Rad21_Rec8_N	STAG	HEAT	TPR	-	HEAT	WAPL	Peptidase_C50	
		Helobdella robusta	-	-	Rad21_Rec8_N	HEAT	HEAT	TPR	-	HEAT	WAPL	Peptidase_C50	
		Daphnia pulex	-	SMC_hinge/SMC_N	Rad21_Rec8/Rad21_Rec8_N	HEAT	HEAT	TPR	-	HEAT	WAPL	Peptidase_C50	
	**Capsaspora**	***Capsaspora owczarzaki***	-	SMC_hinge/SMC_N	Rad21_Rec8/Rad21_Rec8_N	STAG	HEAT	TPR	-	HEAT	WAPL	Peptidase_C50	
	**Fungi**	***Cryptococcus neoformans***	SMC_hinge/SMC_N	SMC_hinge/SMC_N	Rad21_Rec8/Rad21_Rec8_N	STAG	HEAT		-	HEAT	WAPL	Peptidase_C50	
		Ustilago maydis	SMC_hinge/SMC_N	-	Rad21_Rec8/Rad21_Rec8_N	HEAT	HEAT			HEAT	WAPL	Peptidase_C50	
		Aspergillus fumigatus	-	-	Rad21_Rec8/Rad21_Rec8_N	STAG	HEAT	TPR	-	HEAT	WAPL	Peptidase_C50	
		Schizosaccharomyces pombe	SMC_hinge/SMC_N	-	Rad21_Rec8/Rad21_Rec8_N	STAG	HEAT	-	-	HEAT	WAPL	Peptidase_C50	-
		Saccharomyces cerevisiae	-	-	Rad21_Rec8/Rad21_Rec8_N	STAG	HEAT	-	-	HEAT	-	Peptidase_C50	-
		Neurospora crassa	SMC_hinge/SMC_N	SMC_N	Rad21_Rec8/Rad21_Rec8_N	HEAT	HEAT	-		HEAT	WAPL	Peptidase_C50	
		Encephalitozoon cuniculi	SMC_hinge/SMC_N	SMC_hinge/SMC_N		-						Peptidase_C50	
		Enterocytozoon bieneusi H348	-									Peptidase_C50	
		Nosema ceranae BRL01		SMC_hinge/SMC_N		-						Peptidase_C50	
		Encephalitozoon intestinalis ATCC 50506	SMC_hinge/SMC_N	SMC_hinge/SMC_N		-						Peptidase_C50	
		Batrachochytrium dendrobatidis	SMC_hinge/SMC_N	SMC_hinge/SMC_N	Rad21_Rec8_N	STAG	HEAT		-	HEAT	WAPL	Peptidase_C50	
		Spizellomyces punctatus	-	SMC_N	Rad21_Rec8/Rad21_Rec8_N	HEAT	HEAT	-	-	HEAT	WAPL	Peptidase_C50	
**Apusozoa**		***Thecamonas trahens***	SMC_hinge/SMC_N	SMC_hinge/SMC_N	Rad21_Rec8/Rad21_Rec8_N	HEAT	HEAT		-			Peptidase_C50	
**Amoebozoa**		***Dictyostelium discoideum***	-	SMC_hinge/SMC_N	Rad21_Rec8/Rad21_Rec8_N	STAG	HEAT		-	HEAT		Peptidase_C50	
		Entamoeba histolytica	SMC_hinge/SMC_N	-	Rad21_Rec8/Rad21_Rec8_N							Peptidase_C50	
**Excavata**	**Metamonada**	***Giardia intestinalis***	SMC_hinge/SMC_N	SMC_hinge/SMC_N								Peptidase_C50	
		Trichomonas vaginalis	-	-	Rad21_Rec8/Rad21_Rec8_N	HEAT	HEAT			HEAT		Peptidase_C50	
	**Euglenozoa**	***Leishmania major***	SMC_hinge/SMC_N	SMC_N	Rad21_Rec8/Rad21_Rec8_N	STAG			-	HEAT		Peptidase_C50	
		Leishmania infantum	SMC_hinge/SMC_N	-	Rad21_Rec8/Rad21_Rec8_N	STAG			-	HEAT		Peptidase_C50	
		Trypanosoma cruzi	SMC_hinge/SMC_N	SMC_N	Rad21_Rec8/Rad21_Rec8_N	STAG			-	HEAT		Peptidase_C50	
		Trypanosoma brucei	SMC_hinge/SMC_N	SMC_hinge/SMC_N	Rad21_Rec8/Rad21_Rec8_N	STAG			-	HEAT		Peptidase_C50	
	**Heterolobosoa**	***Naegleria gruberi***	-	-	Rad21_Rec8/Rad21_Rec8_N	HEAT	HEAT		-	HEAT		Peptidase_C50	
**Alveolata**	**Ciliata**	***Tetrahymena thermophila***	-	-								Peptidase_C50	
		Paramecium tetraurelia	SMC_hinge/SMC_N	-								Peptidase_C50	
		Oxytricha trifallax	SMC_N	-	-								
	**Apicomplexa**	***Plasmodium yoelii***	-	SMC_hinge/SMC_N		STAG						Peptidase_C50	
		Plasmodium falciparum	SMC_hinge/SMC_N	SMC_hinge/SMC_N		STAG						Peptidase_C50	
		Cryptosporidium hominis	SMC_hinge/SMC_N	SMC_N	Rad21_Rec8/Rad21_Rec8_N							Peptidase_C50	
		Babesia bovis	SMC_hinge/SMC_N	SMC_hinge/SMC_N		STAG							
		Theileria annulata	-			VAR1							
		Toxoplasma gondii	SMC_hinge/SMC_N	SMC_hinge/SMC_N								Peptidase_C50	
**Heterokonta**	**Blastocystae**	***Blastocystis hominis***	SMC_N	-								Peptidase_C50	
	**Phaeophyceae**	***Ectocarpus siliculosus***	SMC_hinge/SMC_N	-	Rad21_Rec8_N	HEAT	HEAT		-	HEAT		Peptidase_C50	
	**Oomycota**	***Phytophthora ramorum***	-	SMC_hinge/SMC_N	Rad21_Rec8/Rad21_Rec8_N	HEAT	HEAT		-	-			
		Phytophthora infestans	SMC_hinge/SMC_N	SMC_hinge/SMC_N	Rad21_Rec8/Rad21_Rec8_N	HEAT	HEAT		-	HEAT		Peptidase_C50	
	**Bacillariophyta**	***Thalassiosira pseudonana***	SMC_hinge/SMC_N		Rad21_Rec8_N		HEAT		-	HEAT		Peptidase_C50	
		Phaeodactylum tricornutum	SMC_hinge/SMC_N	SMC_hinge/SMC_N	Rad21_Rec8/Rad21_Rec8_N		HEAT		-			Peptidase_C50	
		Aureococcus anophagefferens	SMC_hinge/SMC_N	SMC_hinge/SMC_N	Rad21_Rec8_N	HEAT	-					Peptidase_C50	
**Plantae**	**Viridiplantae**	***Oryza sativa***	SMC_hinge/SMC_N	-	Rad21_Rec8/Rad21_Rec8_N	HEAT	HEAT	-	-	HEAT	WAPL	Peptidase_C50	
		Arabidopsis thaliana	-	-	Rad21_Rec8/Rad21_Rec8_N	STAG	HEAT	TPR	-	HEAT	WAPL	Peptidase_C50	
		Chlamydomonas reinhardtii	SMC_hinge/SMC_N	SMC_N	Rad21_Rec8_N	STAG	-			HEAT		Peptidase_C50	
		Ostreococcus tauri	-	SMC_hinge/SMC_N	Rad21_Rec8/Rad21_Rec8_N	HEAT	HEAT	TPR	-	HEAT	WAPL	Peptidase_C50	
		Ostreococcus lucimarinus	SMC_hinge/SMC_N	SMC_hinge/SMC_N	Rad21_Rec8/Rad21_Rec8_N	HEAT	HEAT	TPR	-	HEAT	WAPL	Peptidase_C50	
		Physcomitrella patens	SMC_hinge/SMC_N	XP_001784554	Rad21_Rec8_N	HEAT		TPR	-	HEAT	WAPL	Peptidase_C50	
		Chlorella vulgaris	-	-	Rad21_Rec8/Rad21_Rec8_N	HEAT	HEAT		-	HEAT	-	Peptidase_C50	
		Micromonas pusilla	-	SMC_hinge/SMC_N	Rad21_Rec8/Rad21_Rec8_N	HEAT	HEAT	-	-	HEAT			
		Selaginella moellendorffii	-	SMC_hinge/SMC_N	Rad21_Rec8_N	HEAT	HEAT	TPR	-	HEAT	WAPL	Peptidase_C50	
	**Rhodophyta**	***Cyanidioschyzon merolae***	SMC_hinge/SMC_N	SMC_hinge/SMC_N	Rad21_Rec8/Rad21_Rec8_N	HEAT			-			Peptidase_C50	
		Galdieria sulphuraria	SMC_hinge/SMC_N	-	?	HEAT	?		-	HEAT		Peptidase_C50	
**Haptophyta**		***Emiliania huxleyi***	-	SMC_N	Rad21_Rec8/Rad21_Rec8_N	HEAT	HEAT		-	-		Peptidase_C50	
